# Membrane-To-Nucleus Signaling Links Insulin-Like Growth Factor-1- and Stem Cell Factor-Activated Pathways

**DOI:** 10.1371/journal.pone.0076822

**Published:** 2013-10-07

**Authors:** Yujiro Hayashi, David T. Asuzu, Simon J. Gibbons, Kirsten H. Aarsvold, Michael R. Bardsley, Gwen A. Lomberk, Angela J. Mathison, Michael L. Kendrick, K. Robert Shen, Takahiro Taguchi, Anu Gupta, Brian P. Rubin, Jonathan A. Fletcher, Gianrico Farrugia, Raul A. Urrutia, Tamas Ordog

**Affiliations:** 1 Enteric Neuroscience Program, Department of Physiology and Biomedical Engineering, Mayo Clinic, Rochester, Minnesota, United States of America; 2 Gastroenterology Research Unit, Division of Gastroenterology and Hepatology, Mayo Clinic, Rochester, Minnesota, United States of America; 3 Department of Surgery, Mayo Clinic, Rochester, Minnesota, United States of America; 4 Division of Human Health and Medical Science, Graduate School of Kuroshio Science, Kochi University, Nankoku, Kochi, Japan; 5 Departments of Anatomic Pathology and Molecular Genetics, Lerner Research Institute and Taussig Cancer Center, Cleveland Clinic, Cleveland, Ohio, United States of America; 6 Department of Pathology, Brigham and Women’s Hospital and Harvard Medical School, Boston, Massachusetts, United States of America; 7 Center for Individualized Medicine, Mayo Clinic, Rochester, Minnesota, United States of America; University of Pittsburgh Cancer Institute, United States of America

## Abstract

Stem cell factor (mouse: Kitl, human: KITLG) and insulin-like growth factor-1 (IGF1), acting via KIT and IGF1 receptor (IGF1R), respectively, are critical for the development and integrity of several tissues. Autocrine/paracrine KITLG-KIT and IGF1-IGF1R signaling are also activated in several cancers including gastrointestinal stromal tumors (GIST), the most common sarcoma. In murine gastric muscles, IGF1 promotes Kitl-dependent development of interstitial cells of Cajal (ICC), the non-neoplastic counterpart of GIST, suggesting cooperation between these pathways. Here, we report a novel mechanism linking IGF1-IGF1R and KITLG-KIT signaling in both normal and neoplastic cells. In murine gastric muscles, the microenvironment for ICC and GIST, human hepatic stellate cells (LX-2), a model for cancer niches, and GIST cells, IGF1 stimulated Kitl/KITLG protein and mRNA expression and promoter activity by activating several signaling pathways including AKT-mediated glycogen synthase kinase-3β inhibition (GSK3i). GSK3i alone also stimulated Kitl/KITLG expression without activating mitogenic pathways. Both IGF1 and GSK3i induced chromatin-level changes favoring transcriptional activation at the *Kitl* promoter including increased histone H3/H4 acetylation and H3 lysine (K) 4 methylation, reduced H3K9 and H3K27 methylation and reduced occupancy by the H3K27 methyltransferase EZH2. By pharmacological or RNA interference-mediated inhibition of chromatin modifiers we demonstrated that these changes have the predicted impact on *KITLG* expression. KITLG knock-down and immunoneutralization inhibited the proliferation of GIST cells expressing wild-type KIT, signifying oncogenic autocrine/paracrine KITLG-KIT signaling. We conclude that membrane-to-nucleus signaling involving GSK3i establishes a previously unrecognized link between the IGF1-IGF1R and KITLG-KIT pathways, which is active in both physiologic and oncogenic contexts and can be exploited for therapeutic purposes.

## Introduction

Stem cell factor (mouse: Kitl; human: KITLG) is the natural ligand of the type 3 receptor tyrosine kinase (RTK) KIT. Kitl/KITLG is widely expressed in stromal tissues and is critical for the differentiation, proliferation, migration, survival and functional activation of germinal, erythroid and mast cells and melanocytes [1], as well as interstitial cells of Cajal (ICC), gastrointestinal mesenchymal cells that generate electrical rhythmicity and mediate nerve-smooth muscle interactions [2]. Kitl/KITLG is a non-covalent homodimer and exists as a membrane-bound or locally secreted peptide [3]. A 164-amino-acid secreted isoform results from proteolytic cleavage of the 248-amino-acid, transmembrane peptide (“soluble” isoform; Kitl^248^/KITLG^248^) at a site encoded within exon 6. A 220-amino-acid isoform, which only generates secreted peptide at a slow rate, is produced from an alternatively spliced transcript lacking exon 6 (“membrane-bound” isoform; Kitl^220^/KITLG^220^) [3]. Autocrine/paracrine activation of KIT signaling by KITLG plays a role in several tumors and hematologic malignancies [1]. In other cancers including the majority (75-80%) of gastrointestinal stromal tumors (GIST), which originate from cells of the ICC lineage [4,5], KIT signaling is constitutively active due to oncogenic mutations [6]. GIST lacking mutated *KIT* may harbor activating mutations in PDGF receptor α (*PDGFRA*) [7] or have neither *KIT* nor *PDGFRA* mutations (“wild-type (WT)” GIST) [6]. KIT/PDGFRA inhibitors such as imatinib mesylate are the mainstay of medical treatment for advanced GIST but they are not curative due in part to secondary mutations interfering with drug action [6] or lack of dependence of cancer-initiating cells on KIT/PDGFRA signaling [8]. Since imatinib preferentially targets mutant receptors [6], reduced drug responsiveness [9,10] and aggressive GIST behavior [11] may also reflect activation of WT KIT expressed in the majority of GIST by KITLG originating from the circulation, the tumor cells, or their niche [9,11-13]. However, direct evidence of KITLG-driven GIST cell proliferation is lacking.

Similarly to KIT, PDGFRA and their ligands, insulin-like growth factor (IGF)-1 receptor (IGF1R), a type 2 RTK, and its ligands IGF1 and IGF2 play critical roles in normal growth and development, as well as in cellular stress, aging and cancer by stimulating protein synthesis and the cell cycle [14,15]. IGF1R is expressed and activated in some GIST [16] and is overexpressed in WT GIST [16,17]. Via an autocrine loop, IGF1 stimulates the growth and survival of gastrointestinal smooth muscle cells [18-20], and, thereby, promotes the differentiation of ICC [21] by increasing Kitl availability in their microenvironment [22]. IGF1 also activates gene transcription e.g. via p44/p42 mitogen-activated protein kinase (ERK1/2 MAPK) signaling [15] or by promoting the nuclear translocation and binding to the chromatin of IGF1R [23]. Together, these observations suggest that there may be cooperation between the IGF1**-**IGF1R and KITLG**–**KIT signaling pathways both in normal tissues and certain cancers including GIST; these interactions may be mediated by epigenetic control of gene transcription; and increased Kitl/KITLG expression may result in autocrine/paracrine stimulation of proliferation in cells expressing WT KIT. Here, we tested these hypotheses by investigating the effects of IGF1 on endogenous *Kitl/KITLG* expression and the underlying epigenetic mechanisms and signaling pathways in IGF1R-expressing cells and tissues including gastric smooth muscles [18-20,22], the natural microenvironment for ICC and GIST; in human GIST cells [10,16], and in LX-2 human hepatic stellate cells [24], a model for cancer niches [25]. Our findings indicate that IGF1 stimulates *KITLG* transcription by inducing coordinated chromatin modifications in part via glycogen synthase kinase (GSK)-3β inhibition. We also provide evidence supporting KITLG-mediated autocrine/paracrine stimulation of cell proliferation in GIST cells expressing WT KIT.

## Materials and Methods

### Ethics statements

Mice were maintained and the experiments were performed in accordance with the National Institutes of Health Guide for the Care and Use of Laboratory Animals. The protocol was approved by the Institutional Animal Care and Use Committee of the Mayo Clinic (A64812). De-identified human gastric tissues used for the preparation of primary cell cultures were obtained as surgical waste tissue from patients undergoing bariatric surgery with the approval of the Mayo Clinic Institutional Review Board (07-003371). The Mayo Clinic Institutional Review Board waived the need for written informed consent from the participants.

### Tissue preparation

BALB/c mice aged 14-16 days were obtained from breeder pairs purchased from Harlan Laboratories (Madison, WI). LONG R^3^-recombinant human IGF1 (LR3-rhIGF1; Research Peptides, Orlando, FL) was administered by a single i.p. injection. Mice were anesthetized with isoflurane (AErrane; Baxter Healthcare, Deerfield, IL) inhalation and killed by decapitation. Intact gastric corpus and antrum *tunica muscularis* tissues were dissected as described [22] and used either as organotypic cultures [22] or primary cell cultures [26]. Primary cell cultures were prepared from human *tunica muscularis* as described for murine tissues [26].

### Tissue culturing and drug treatment

Gastric *tunica muscularis* organotypic cultures were maintained for up to 24 h. Pharmacological agents were applied as indicated in the Results and Figures. In some experiments tissues were preincubated for 3 h with specific, cell-permeable inhibitors or dimethyl sulfoxide (DMSO) vehicle before exposure to rhIGF1 for 18 h in the continuing presence of the inhibitors. Pharmacological agents included rhIGF1, the ATP-competitive GSK3α/β inhibitors SB415286 and SB216763 [27], the non-ATP-competitive GSK3α/β inhibitor TDZD-8 [28], adenosine dialdehyde (Adox) [29], an inhibitor of S-adenosylhomocysteine hydrolase and indirect inhibitor of S-adenosyl-methionine-dependent methylation reactions including trimethylation of lysine 27 of histone 3 (H3K27me3) by the polycomb repressive complex 2 (PRC2) member enhancer of zeste homolog 2 (EZH2) (Sigma-Aldrich, St. Louis, MO); Tyrphostin AG1024, a specific inhibitor of IGF1 and insulin RTK activity [30]; AKT Inhibitor X (10-(4'-(N-diethylamino)butyl)-2-chlorophenoxazine, HCl), an inhibitor of AKT phosphorylation, in-vitro kinase activity and IGF1-induced nuclear translocation [31]; rapamycin, inhibitor of mechanistic target of rapamycin (MTOR) complex 1 and ribosomal p70 S6 kinase (p70S6K) phosphorylation [20]; and PD98059, inhibitor of MEK1/2 ERK MAPK kinases [32] (EMD Chemicals, Inc., Gibbstown, NJ).

### Cell cultures and antibody/drug treatment

Primary cell cultures prepared from human or murine gastric smooth musculature were maintained as described previously [26]. Stromal cells derived from the fetal hematopoietic microenvironment of Kitl-deficient *Sl/Sl* ^4^ mice, *Sl/Sl* ^4^ stromal cells genetically modified to express full-length murine Kitl (*Sl/Sl* ^*4*^-Kitl^248^) (generously donated by Dr. David Williams, Indiana University School of Medicine, Indianapolis, IN) [3], human LX-2 spontaneously immortalized hepatic stellate cells (kindly provided by Dr. Scott Freeman, Mount Sinai School of Medicine, New York, NY) [33], and human GIST-T1 cells derived from metastatic pleural tumor from a gastric GIST containing a heterozygous deletion of 57 bases in exon 11 juxtamembrane domain of *KIT* (contributed by Dr. Takahiro Taguchi) [34] were cultured in high-glucose Dulbecco’s modified Eagle’s medium (DMEM; Gibco, Life Technologies, Carlsbad, CA) containing 10% fetal bovine serum (FBS) and 1% antibiotic-antimycotic (Gibco) at 37 °C in the presence of 5% CO_2_. GIST-T1-5R cells derived from GIST-T1 cells by prolonged in vitro exposure to imatinib and carrying a secondary, imatinib-resistant T670I mutation in exon 14 (contributed by Dr. Anu Gupta and Dr. Brian P. Rubin) were propagated in the presence of 1 µM imatinib mesylate (LC Laboratories, Woburn, MA). Imatinib was removed from the culture media 4 days prior to the experiments. GIST882 human cells from a primary GIST containing a homozygous *KIT* exon 13 missense mutation leading to K642E substitution in the first part of the split tyrosine kinase domain (contributed by Dr. Jonathan Fletcher) [35] were also cultured in high-glucose DMEM containing 10% FBS and 1% antibiotic-antimycotic but were maintained in a milieu of 4% O_2_, 5% CO_2_ and 91% N_2_. GIST48B KIT^low/–^ human cells derived from GIST48 cells containing *KIT* exon 11 (homozygous V560D: imatinib-sensitive) and exon 17 phosphotransferase domain (heterozygous D820A: imatinib-resistant) mutations by prolonged heat shock protein 90 inhibition (contributed by Dr. Jonathan Fletcher) [36] were maintained with Iscove’s DMEM (high glucose) containing 15% FBS, 1% l-glutamine and 1% antibiotic-antimycotic (Gibco) at 37 °C in the presence of 5% CO_2_. LX-2, GIST-T1 and GIST882 cells have previously been demonstrated to express IGF1R [16,24]; results showing IGF1R α and β chain expression in GIST48B cells and IGF1 secretion by LX-2, GIST-T1, GIST882 and GIST48B cells are shown in Figure S1F-G. The role of endogenous KITLG in the proliferation of GIST-T1, GIST-T1-5R, GIST882, GIST48B and LX-2 cells was tested by culturing in the above media in the presence of purified, azide-free goat polyclonal anti-human KITLG antibody (AB-255-NA, R&D Systems, Minneapolis, MN; applied for 4 days at concentrations indicated in the Results), which has been shown to neutralize KITLG-induced proliferation in the TF1 human erythroleukemic cell line [37]. The specificity of KITLG immunoneutralization was verified by preabsorbing the anti-KITLG antibody with rhKITLG (R&D Systems) applied at 10:1 molar ratio overnight at 4 °C. Cell proliferation was assessed by the CellTiter 96^®^ AQ_ueous_ Non-Radioactive Cell Proliferation Assay (Promega, Madison, WI) according to the manufacturer’s protocol. To examine the effects of directly modifying the epigenetic status of chromatin on *KITLG* expression, LX-2 and GIST-T1 cells were treated after 24-h serum starvation with the following specific, cell-permeable drugs or DMSO vehicle for 24-72 h at the concentrations indicated in the text: Adox, BIX-01294, a non-S-adenosyl-methionine analog-based inhibitor of the histone-lysine methyltransferases (HKMT) G9A (EHMT2) and GLP (EHMT1) and the H3K9me1/2 (histone H3 lysine 9 mono/ dimethylation) modification they catalyze [38,39]; chaetocin, an S-adenosyl-methionine-competitive inhibitor of the SUV39 family of H3K9 HKMTs including G9A, GLP and SUV39H1 [39,40]; garcinol, an inhibitor of histone acetyltransferases (HAT) p300 (EP300) and PCAF (KAT2B) (Sigma-Aldrich) [41]; and suberoylanilide hydroxamic acid (SAHA; vorinostat), a class I-II histone deacetylase (HDAC) inhibitor (Santa Cruz Biotechnology, Dallas, TX) [42].

### RNA interference (RNAi)

RNAi against KITLG and heterochromatin protein 1 (HP1) homolog α (CBX5) was performed using Dharmacon ON-TARGET*plus*® SMARTpool® small interfering RNA (siRNA) or corresponding scrambled sequences (25 nM) and DharmaFECT 4 Transfection Reagent (Thermo, Fisher Scientific, Waltham, MA) according to the manufacturer’s protocol. HP1β (CBX1) and HP1γ (CBX3) were targeted with short hairpin RNAs (shRNAs) containing 19-mer antisense sequences (HP1β: GAAAGGGAGATGGGTAGCATC; HP1γ: GCAAATCAAAGAAGAAAAG). The sense-loop-antisense-terminator shRNA template inserts were cloned in under the RNA polymerase III H1 promoter in a bicistronic plasmid assembled to express green fluorescent protein from a CMV promoter. Plasmids were transformed into DH5a competent cells (Invitrogen, Carlsbad, CA), expanded and purified using a Plasmid Maxi Kit (QIAGEN, Germantown, MD). Plasmids (30 µg) were electroporated 3 times into LX-2 cells. Transfection efficacy was estimated after 24 h by fluorescence microscopy; cells were harvested after 72 h. Off-target effects were controlled for by transfecting cells with empty vectors.

*Reverse transcription-polymerase chain reaction (RT-PCR*). *Kitl/KITLG* transcription was monitored by real-time or traditional RT-PCR (see details including controls in ref [43].) using specific, intron-spanning primers published previously [22] or designed for this study (human *KITLG* exons 1-2: forward: TGCGCTCGGGCTACCCAATG; reverse: GCAGATCCCTTCAGTTTTGACGAGAG). Transcriptional quantification was obtained by the ΔΔC_T_ method on a Bio-Rad Laboratories (Hercules, CA) CFX96 real-time PCR detector.

### KITLG transcriptional activity

A human *KITLG* promoter-pGL3b luciferase construct was generated in a two-step process. First, a 1452-bp product of the 5' promoter region (-2120 bp to -669 bp) of *KITLG* was obtained by PCR amplification of human genomic DNA using specific primers. The 5' primer contained a KpnI restriction site at the 5' end for incorporation into the vector. The BglII site contained within the promoter region (-842 bp to -837 bp) was utilized as the 3' restriction site and the 1280-bp KpnI-BglII digestion product was ligated into the KpnI-BglII sites of the pGL3 basic vector (Promega). The 5' *KITLG* promoter sequence was confirmed and, subsequently, the vector was reopened at the BglII site. A 1280-bp product of the 3' *KITLG* promoter region (-873 bp to +407 bp) was amplified by PCR, utilizing human genomic DNA and specific primers with the 3' primer containing a BglII site at the 5' end for incorporation into the vector. Again, the BglII site contained within the promoter region was utilized this time as the 5' restriction site and the 1245 bp BglII-BglII digestion product was ligated into the BglII site of the 5' *KITLG* promoter (-2120 bp to -837 bp)-pGL3b construct. Orientation and the entire promoter sequence was confirmed by sequencing to obtain the full length *KITLG* promoter (-2120 bp to +407 bp)-pGL3b luciferase construct. Transfection of LX-2 and GIST-T1 cells, as well as primary cell cultures prepared from murine gastric smooth muscles was performed as described above. Cells were harvested, lysed and assayed for luciferase activity 48 h after transfection using the Promega Luciferase Assay System.

### Western immunoblotting

Tissue and cell lysates were prepared and subjected to sodium dodecyl sulfate–polyacrylamide gel electrophoresis and immunoblotting as described previously [44] (see antibodies in Table S1). Bound antibodies were visualized using an Odyssey Infrared Imaging System (LI-COR Bioscience, Lincoln, NE) and Bio-Rad Quantity One 4.5.1 software. Protein and phosphoprotein bands of interest were expressed in densitometric units normalized to the loading control (glyceraldehyde-3-phosphate dehydrogenase; Gapdh/GAPDH or β-actin; Actb) and the corresponding total protein, respectively, detected simultaneously in the same sample.

### Chromatin immunoprecipitation (ChIP)

DNA–protein complexes from juvenile murine gastric smooth muscle were cross-linked using fresh 1% formaldehyde (Thermo, Fisher) for 10 minutes, followed by glycine quenching. DNA-protein complexes were sheared on cold water using a Bioruptor sonicator (Diagenode, Denville, NJ) at settings optimized for obtaining DNA fragments ranging from ~150 to 600 bp. Histone and chromatin-binding proteins were purified and immunoprecipitated overnight at 4 °C along with bound DNA fragments using reagents and magnetic beads from a EZ-Magna ChIP™ G Chromatin Immunoprecipitation Kit (Millipore, Billerica, MA) and ChIP-grade antibodies against EZH2, H3K27me3, H3K4me2 (dimethylated lysine 4 of histone 3), H3K9me2, H3K9me3, H3K9ac (acetylated lysine 9 of histone 3) and H4ac (acetylated histone 4) (Table S2). The ability of these antibodies to enrich target DNA was verified by PCR and antibodies against RNA polymerase II (positive control), Gapdh (negative control) and “non-immune” mouse IgG (negative control). Bound DNA fragments were isolated by proteinase K digestion for 2 hours at 62 °C, and magnetic beads were dissociated by incubation at 95 °C for 10 minutes. Immunoprecipitated DNA fragments were analyzed by quantitative real-time PCR using primers designed to target the mouse *Kitl* promoter (-300 bp to -214 bp): forward: GCTGGTGAGCTTGCTGCGGA; reverse: TGAGGCACCGGGAGTCTCGG. PCR results were quantified by the ΔΔC_T_ method using input DNA as reference and the vehicle (DMSO)-treated samples as control.

### Statistical analyses

Each data point (n) represents one freshly isolated or cultured stomach or a biological replicate experiment in cultured cells. Data are expressed as mean ± standard error of the mean (SEM) or median and interquartile range. Student’s *t* test or Mann-Whitney rank sum test were used for comparing two data sets. Three groups or more were compared by one-way analysis of variance (ANOVA) or ANOVA on ranks followed by multiple comparisons. A probability value of *P*<0.05 was used as a cut-off for statistical significance in all statistical procedures. IC_50_ values were obtained by nonlinear curve fitting applied to dose-response data using the equation library in SigmaPlot 10.0 (Systat Software, Chicago, IL).

## Results

### Kitl/KITLG protein is expressed in IGF target cells and tissues

**Figure 1:**
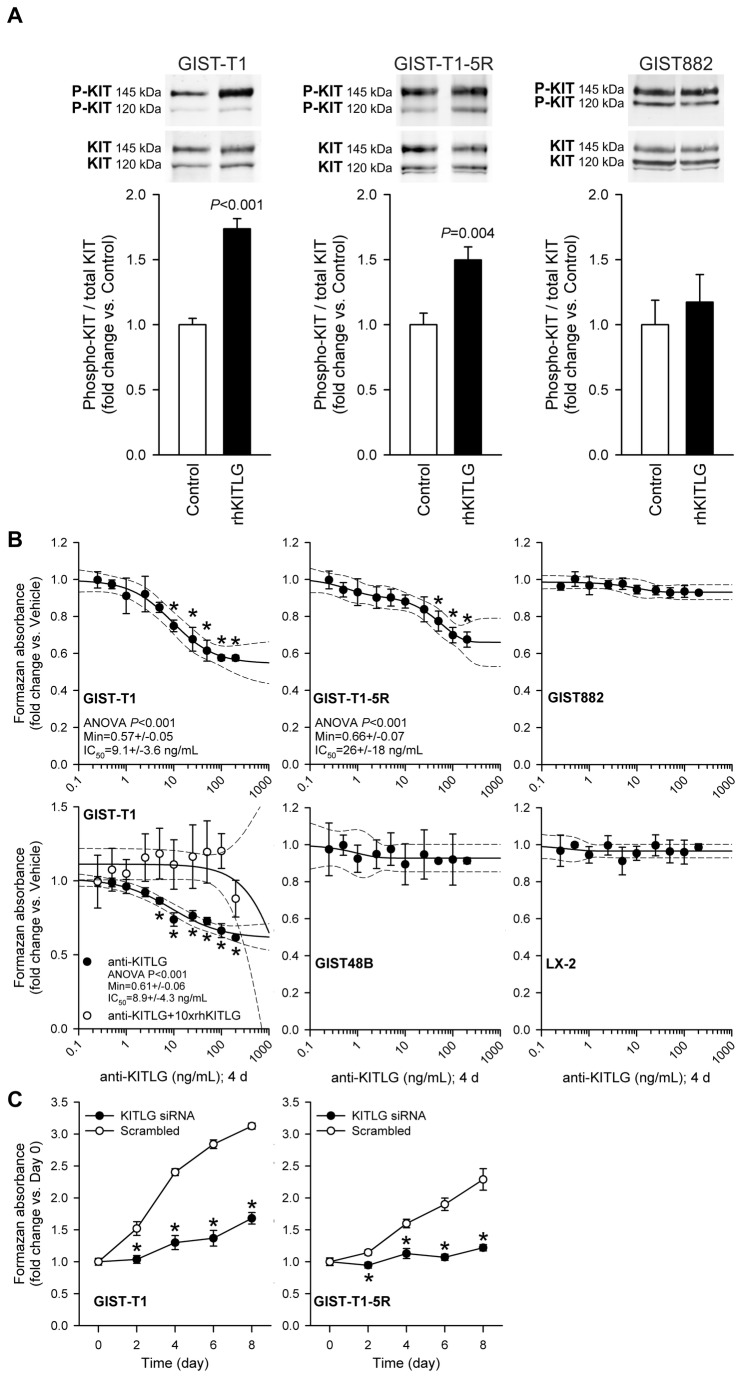
Contribution of KITLG-activated KIT signaling to baseline proliferation of GIST and LX-2 cells. *A*, KIT Y721 phosphorylation was activated by exogenous rhKITLG (100 ng/mL for 10 min following 2 h serum deprivation [13,35]) in GIST-T1 cells containing a heterozygous activating *KIT* mutation (n=4/group) and in GIST-T1-5R cells containing an additional, imatinib-resistant *KIT* mutation (n=6/group), but not in GIST882 cells lacking a WT *KIT* allele (n=5/group). *B*, Culturing with anti-human KITLG neutralizing antibody for 4 days inhibited baseline proliferation of GIST-T1 cells (*P*<0.001; n=3; regression and 95% confidence band are shown as solid and dashed lines, respectively) and GIST-T1-5R cells (*P*<0.001; n=6). The effect of KITLG immunoneutralization on GIST-T1 cells was blocked by preabsorbing the antibody with 10-fold molar excess of rhKITLG (see open symbols in the left panel, second row; n=3/group). KITLG immunoneutralization did not inhibit the proliferation of GIST882 or GIST48B cells lacking a WT *KIT* allele (note that GIST48B cells also express very low to undetectable levels of KIT protein, see Figure S1E) and of LX-2 cells lacking KIT protein (Figure S1E) (n=3/cell line). *C*, Inhibition of the proliferation of GIST-T1 and GIST-T1-5R cells by siRNA-mediated knock-down of *KITLG* (n=4/cell line/group; *P*_GIST-T1_: day 2: 0.008, days 4, 6 and 8: <0.001; *P*_GIST-T1-5R_: day 2: <0.02, day 4: 0.004, days 6 and 8: <0.001).

We investigated Kitl/KITLG protein expression in IGF1R-expressing gastrointestinal smooth muscles [18,20,22], LX-2 hepatic stellate cells [24] and GIST cell lines (see ref [16]. and Figure S1F). In murine gastric muscles, Kitl protein could be readily detected by Western immunoblotting as a ~31-kDa band (Figure S1A) corresponding to uncleaved, cell-associated Kitl^220^ and secreted Kitl produced from the Kitl^248^ isoform [3]. Occasionally, we also detected a 43-kDa band likely representing residual, uncleaved Kitl^248^ and a 21-kDa band, which corresponds to secreted Kitl produced from Kitl^220^ [3]. However, the low abundance of these minor peptides did not allow quantification. In GIST and LX-2 cells, only the 31-kDa KITLG band was detected (Figure S1B). We validated our method in *Sl/Sl* ^4^ fibroblasts lacking full-length Kitl and in *Sl/Sl* ^4^ cells engineered to stably express Kitl^248^ (Figure S1C) or Kitl^220^ (not shown) [3]. KITLG expression was similar in KIT^+^ GIST-T1 cells expressing WT KIT, in KIT^+^ GIST882 and KIT^low/–^ GIST48B cells lacking a WT *KIT* allele, and in KIT^-^ LX-2 cells (Figure 1D-E). These results demonstrate Kitl/KITLG expression in all our models.

### Endogenous KITLG stimulates the proliferation of GIST cells expressing WT KIT

To investigate the role of autocrine/paracrine KITLG–KIT signaling in GIST proliferation, we examined the effect of KITLG immunoneutralization and RNAi-mediated knock-down in GIST cells expressing or lacking WT KIT. Control experiments verified the induction of KIT phosphorylation on Y721, the docking site for the p85 subunit of PI3K [1], by exogenous KITLG in GIST-T1 cells containing a heterozygous activating *KIT* mutation (Figure 1A; see reagent concentrations, exposure times, replicate numbers and other statistical details in the figures and their legends). KIT phosphorylation was also increased, albeit to a lesser degree, in GIST-T1-5R cells, derivatives of GIST-T1 cells containing an additional, imatinib-resistant *KIT* mutation. In contrast, no ligand-dependent KIT phosphorylation was detected in GIST882 cells, which are homozygous for the activating *KIT* ^K642E^ mutation. Culturing with anti-human KITLG neutralizing antibody [37] for 4 days inhibited the proliferation of GIST-T1 cells by ~40% (IC_50_: ~9 ng/mL) and GIST-T1-5R cells by ~34% (IC_50_: ~26 ng/mL) (Figure 1B), whereas the same treatment had no effect on KIT^+^ GIST882 and KIT^low/–^ GIST48B cells lacking WT *KIT* allele or on KIT^-^ LX-2 cells. The effect of KITLG immunoneutralization on GIST-T1 cells was prevented by preabsorbing the antibody with 10-fold molar excess of rhKITLG (Figure 1B). The proliferation of both GIST-T1 and GIST-T1-5R cells could also be inhibited by RNAi targeting *KITLG* (Figure 1C). These findings provide direct evidence that activation of KIT signaling by endogenous KITLG contributes to the proliferation of GIST cells expressing WT receptors.

### IGF1 stimulates Kitl protein expression

**Figure 2:**
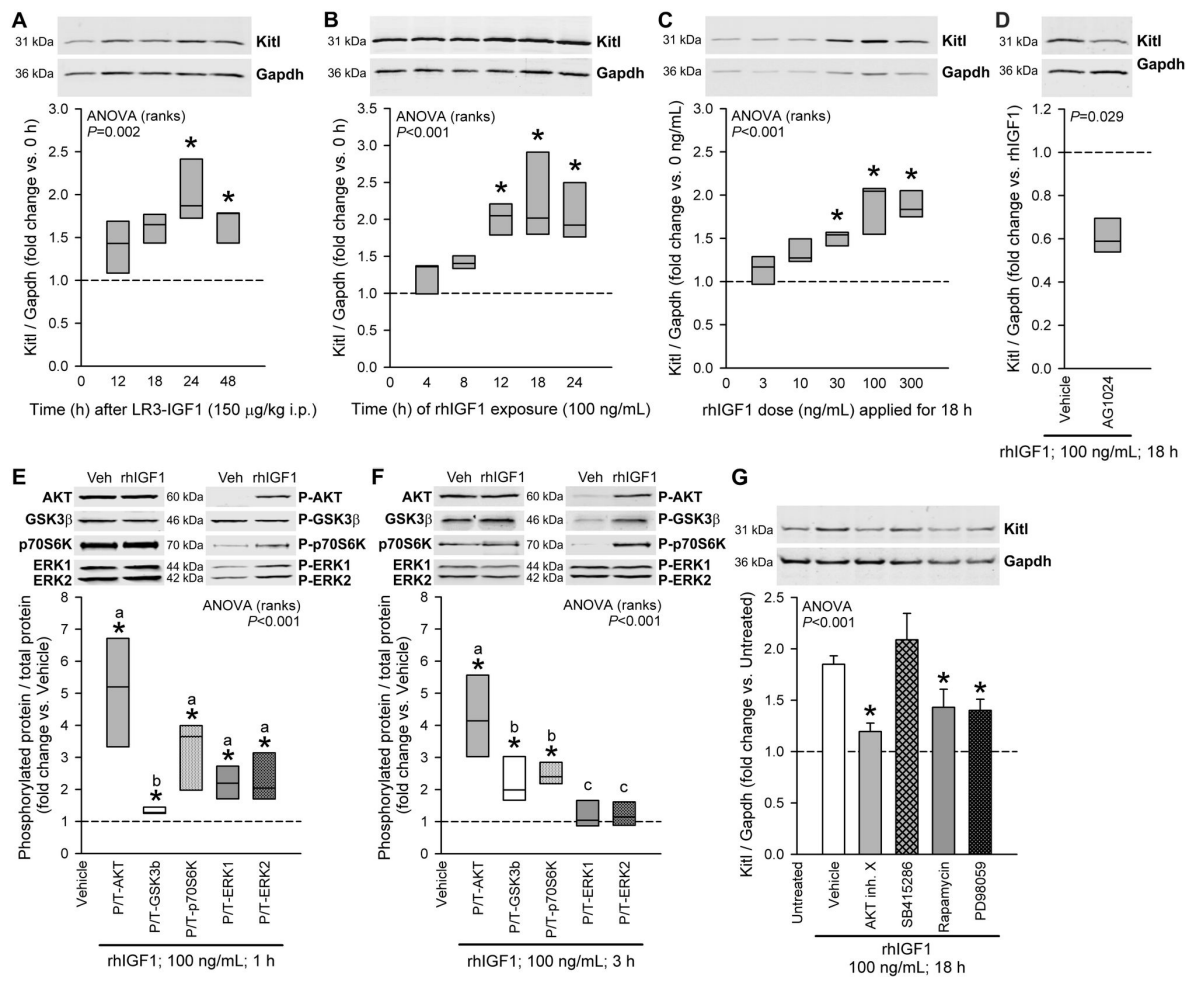
Kitl protein expression is stimulated by IGF1 via multiple IGF1R-activated pathways. *A*, Time-dependent stimulation of Kitl expression in the gastric *tunica* *muscularis* of 14-16-day-old BALB/c mice by the potent IGF1 analog LR3-rhIGF1 administered in a single 150 µg/kg dose i.p.; n=5 mice/group. *B*, Time-dependent stimulation of Kitl expression by 100 ng/mL rhIGF1 in gastric corpus+antrum *tunica* *muscularis* organotypic cultures from 14-16-day-old BALB/c mice; n=4-5/group. *C*, Concentration-dependent stimulation of Kitl protein expression by 18-h rhIGF1 treatment; n=7 organotypic cultures/group. *D*, Blockade of the rhIGF1-induced Kitl expression in organotypic cultures by AG1024 (1 µM), a specific inhibitor of IGF1/insulin receptor tyrosine kinase activity; n=4/group. *E*-*F*, Effects of 1-h (*E*) and 3-h (*F*) rhIGF1treatment on AKT (S473/S474/S472), GSK3β (S9), p70S6K (T389) and ERK1/2 (T202/Y204 and T185/Y187) phosphorylation; n=5 organotypic cultures/group. *G*, Blockade of rhIGF1-induced Kitl expression in organotypic cultures by specific inhibitors of AKT (AKT Inhibitor X; 150 µM), MTOR–p70S6K (rapamycin; 1.5 nM) and MEK–ERK (PD98059; 50 µM); n=5-16/group. The GSK-3 inhibitor SB415286 (30 µM), which is expected to mimic, rather than inhibit, the effect of the IGF1-induced inhibitory phosphorylation of GSK-3, had no significant effect. Kitl and Gapdh or total (T) and phosphorylated proteins (P) were simultaneously detected in the same samples by two-color immunofluorescence (Figure S1). Representative immunoblots show identical areas of the blots imaged at different wavelengths. Box plots show medians and interquartile ranges; bar graphs indicate means±SEM. Data were normalized to the control groups indicated in the panels (dashed lines). Groups marked by asterisk are different from the control group, and groups not sharing the same superscript letter are different from each other (*P*<0.05 by post-hoc multiple comparisons). IGF1 stimulated Kitl expression in gastric smooth muscles in vivo and in vitro in a time- and concentration-dependent manner by activating IGFR1 and the AKT–GSK3, MTOR–p70S6K and ERK MAPK pathways.

To investigate the direct effects of IGF1 on Kitl expression, we first administered 150 µg/kg LR3-rhIGF1, a potent IGF1 analog with reduced affinity for IGF-binding proteins, to 14-16-day-old BALB/c mice in a single i.p. injection and measured Kitl protein in the gastric *tunica muscularis* by Western blotting. Kitl expression increased in a time-dependent manner, with maximum effect occurring at 24 h (Figure 2A). In short-term cultures of intact gastric corpus+antrum muscles from 14-16-day-old BALB/c mice, 100 ng/mL rhIGF1 caused maximum stimulation of Kitl protein between 12 and 24 h (Figure 2B). This effect was dose-dependent, plateauing between 100 and 300 ng/mL (Figure 2C). The upregulation of Kitl expression seen in response to 100 ng/mL rhIGF1 applied for 18 h was also verified using Actb as loading control (Figure S2). Tyrphostin AG1024, a specific inhibitor of IGF1R and insulin RTK activity [30], reduced the rhIGF1 effect on Kitl (Figure 2D) indicating that it was likely mediated by IGF1R or insulin receptor/IGF1R heterodimers [15].

### Multiple signaling pathways mediate IGF1-induced Kitl protein expression

To investigate the mechanisms of IGF1-induced Kitl expression, we first detected the phosphorylation of key IGF1 signaling intermediates [19,20] in murine gastric muscles stimulated by exogenous IGF1 (Figure 2E-F). rhIGF1 elicited time-dependent increase in the phosphorylation of AKT, GSK3β, p70S6K and ERK1/2: After 1 h, we detected increased activating phosphorylation on S473/S474/S472 of AKT isoforms, p70S6K phosphorylation on T389 [19], elevated ERK1 and ERK2 phosphorylation on T202/Y204 and T185/Y187, respectively, and increased inhibitory phosphorylation on S9 of GSK3β [45] (Figure 2E). After 3 h, ERK1/2 phosphorylation returned to baseline but phosphorylated AKT, p70S6K and GSK3β remained elevated (Figure 2F). We then probed the contribution of these intermediates to IGF1-induced Kitl expression by using pathway-specific inhibitors (Figure 2G): rhIGF1-stimulated Kitl expression was reduced by AKT Inhibitor X [31]; by rapamycin, which selectively inhibits the activation of p70S6K by MTOR complex 1 [19]; and by PD98059, a selective MEK inhibitor [32]. SB415286, a selective competitive inhibitor of ATP binding to GSK3α/β and functional mimic of AKT-mediated GSK-3 phosphorylation and inactivation [27], only minimally increased Kitl expression beyond the near-maximal stimulation caused by IGF1. These results indicate cooperation among several major IGF1 intermediate pathways in stimulation of Kitl expression.

### GSK-3 inhibition (GSK3i) stimulates Kitl expression without directly activating pathways involved in cell growth and proliferation

**Figure 3:**
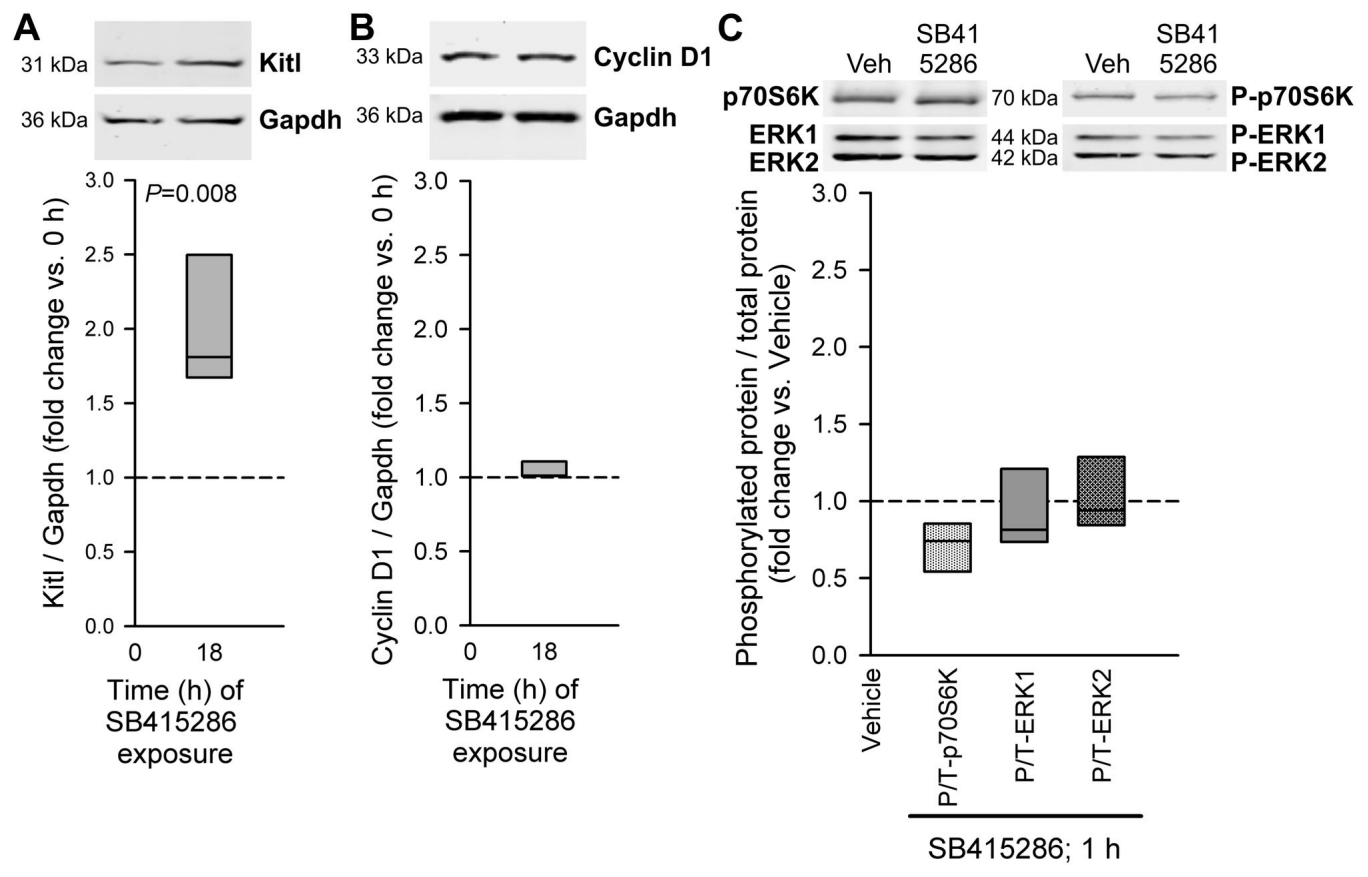
GSK3i stimulates Kitl expression without activating cyclin D1 expression and p70S6K and ERK1/2 phosphorylation. The GSK3α/β inhibitor SB415286 was applied to organotypic cultures of gastric corpus+antrum muscles from of 14-16-day-old BALB/c mice at 30 µM. *A*, Effect of 18-h application of SB415286 on Kitl expression; n=5/group. *B*, Effect of the same treatment on cyclin D1 expression; n=3/group. *C*, Effects of 1-h exposure to SB415286 on p70S6K and ERK1/2 phosphorylation; n=3/group. See Figure 2 for further details.

To better understand the role of GSK3i in the stimulation of Kitl expression, we evaluated the effects of SB415286 in the absence of exogenous IGF1 in isolated murine gastric muscles. Under this condition, SB415286 stimulated Kitl expression (Figure 3A). Despite mimicking the effect of IGF1 on Kitl, SB415286 did not stimulate the expression of cyclin D1, a key mediator of IGF1-induced cell cycle progression [15] (Figure 3B), or the phosphorylation of p70S6K and ERK1/2 (Figure 3C) at time points when IGF1 effects on these parameters were prominent. We obtained similar results with SB216763, another ATP-competitive GSK3α/β inhibitor and TDZD-8, a non-ATP-competitive GSK3α/β inhibitor (Figures S3 and S4). Thus, GSK3i alone specifically stimulates Kitl expression offering a pharmacological approach to increase Kitl/KITLG levels in gastrointestinal muscles without reproducing IGF1’s actions promoting cellular stress, aging and cancer [14,15].

### IGF1 and GSK3i activate Kitl/KITLG transcription

**Figure 4:**
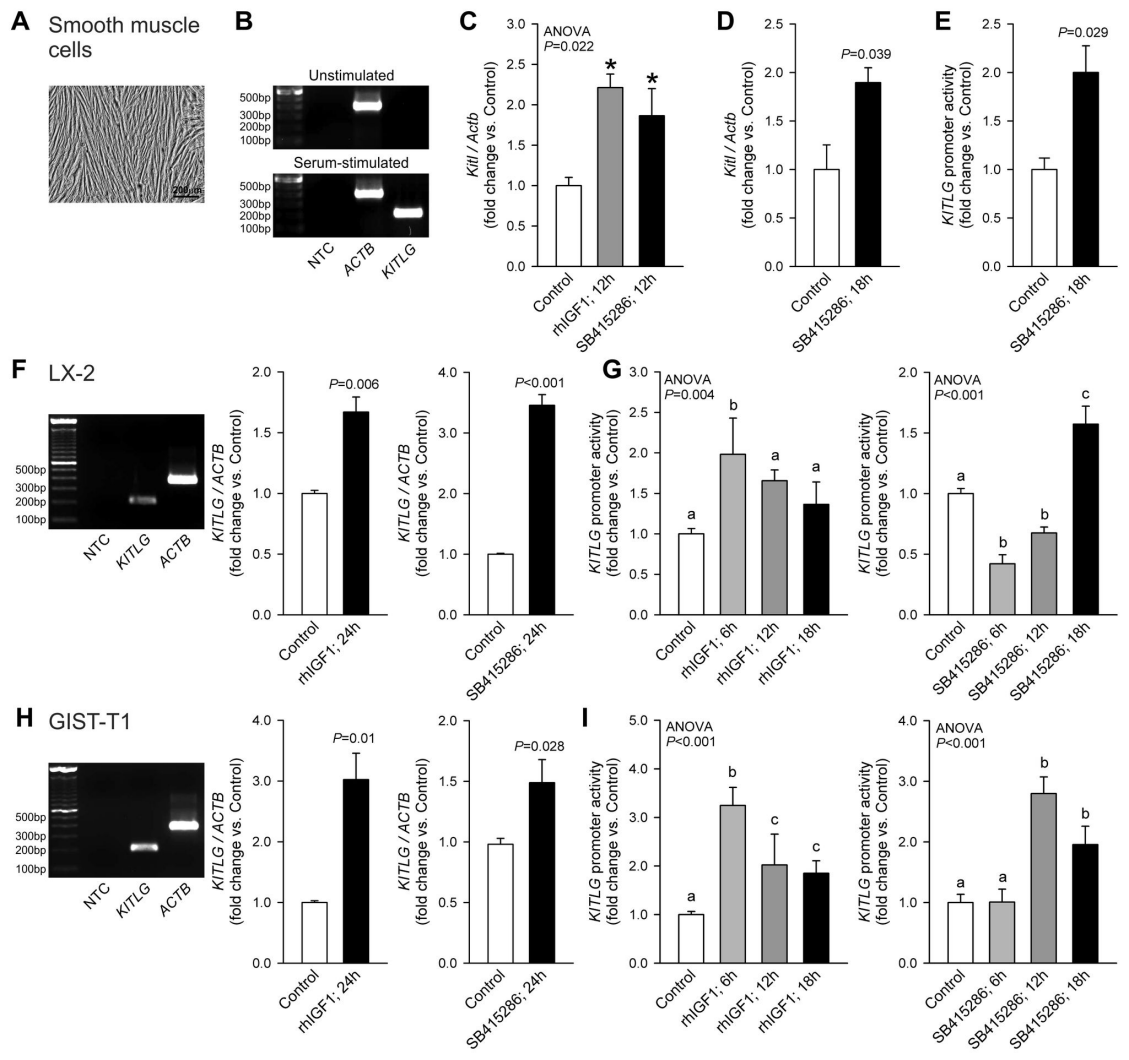
IGF1 and GSK3i stimulate *Kitl/KITLG* transcription. Results obtained in murine and human smooth muscle cells (*A*-*E*), human LX-2 stellate cells (*F*-*G*) and human GIST-T1 cells (*H*-*I*) are shown. *A*, Hoffman modulation contrast image of primary human gastric smooth muscle cells. *B*, *KITLG* mRNA (total: soluble+membrane-bound) was readily detectable in primary human smooth muscle cells (passage 3) maintained with Smooth Muscle Growth Medium-2 containing insulin, hFGF-B, hEGF and 5% FBS (Lonza) but not in 24-h growth factor- and serum-deficient basal medium. *C*, Both IGF1 (100 ng/mL) and the GSK3α/β inhibitor SB415286 (30 µM) stimulated *Kitl* expression in murine gastric *tunica* *muscularis* organotypic cultures (n=3/group). *D*-*E*, SB415286 stimulated endogenous *Kitl* expression in murine primary gastric smooth muscle cells (*D*; n=3/group) and *KITLG* transcriptional activity in the same cell type transfected with a *KITLG* promoter (-2120 bp to +407 bp)-pGL3 luciferase construct (*E*; n=3/group). IGF1 (100 ng/mL; n=3/group) and SB415286 (30 µM; n=6/group) also increased endogenous *KITLG* mRNA expression in LX-2 (*F*) and GIST-T1 cells (*H*) and stimulated *KITLG* promoter activity in a time-dependent fashion (LX-2: n=6-9/group; *G*; GIST-T1: n=3-11/group; *I*). Groups marked by asterisk are different from the control group, and groups not sharing the same superscript letter are different from each other (*P*<0.05 by post-hoc multiple comparisons).

Next, we investigated the role of gene transcription in the IGF1 and GSK3i effects. In primary human smooth muscle cells (Figure 4A), *KITLG* mRNA was expressed in a serum-dependent fashion (Figure 4B). In intact murine smooth muscles, 12-h exposure to rhIGF1 or SB415286 upregulated *Kitl* mRNA (Figure 4C). In primary murine smooth muscle cells, SB415286 stimulated endogenous *Kitl* expression (Figure 4D) and activated transcription from an episomally expressed human *KITLG* promoter- luciferase construct (Figure 4E). In LX-2 and GIST-T1 cells, both rhIGF1 and SB415286 increased *KITLG* mRNA (Figure 4F, H), although SB415286 was more effective in LX-2 cells and IGF1 had a greater effect in GIST-T1 cells. *KITLG* promoter activity was also increased by both IGF1 and SB415286 (Figure 4G, I). In both cell lines, IGF1 displayed more rapid action on *KITLG* promoter activity than SB415286. Nevertheless, these results indicate that the stimulation of Kitl protein expression by IGF1 and GSK3i primarily occurs at the transcriptional level and led us to investigate the nature of chromatin remodeling events that account for this effect.

### Coordinated chromatin modifications underlie the activation of Kitl/KITLG transcription by IGF1 and GSK3i

**Figure 5:**
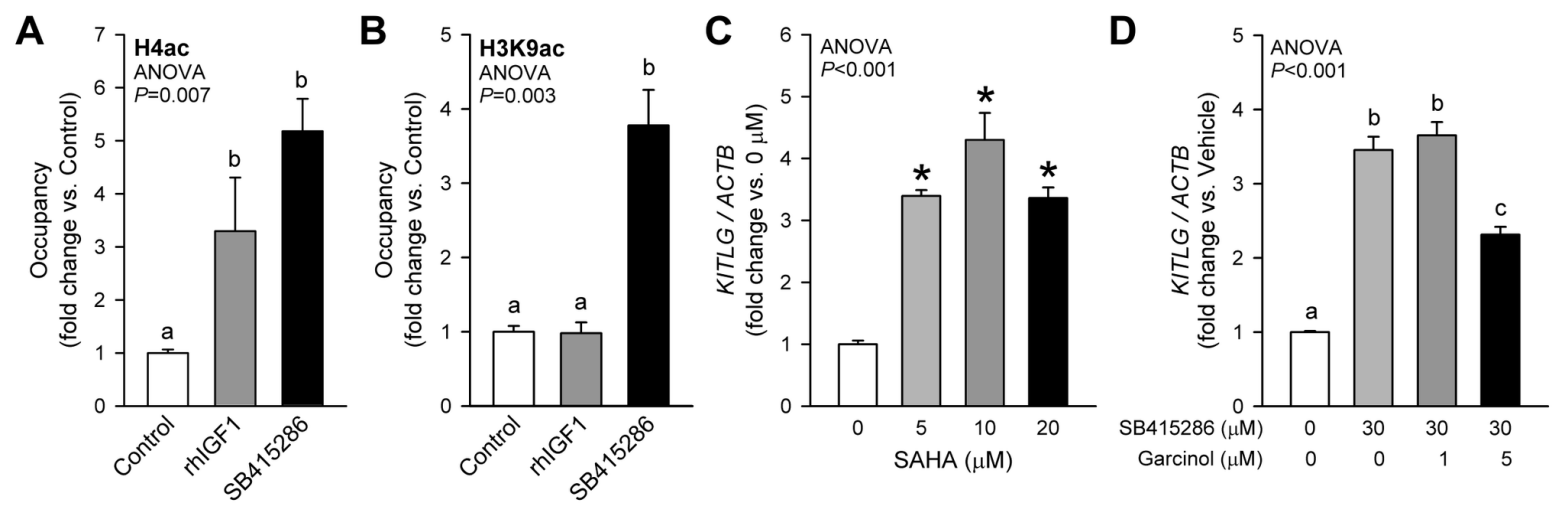
Role of histone acetylation in the activation of *Kitl/KITLG* transcription by IGF1 and GSK3i. *A*, Increased occupancy of the *Kitl* core promoter region by H4ac in response to 6-h rhIGF1 (100 ng/mL) and SB415286 (30 µM) treatment in murine gastric smooth muscles. Only SB415286 increased occupancy by H3K9ac (*B*). Representatives of two independent experiments, each performed in triplicates, are shown. *C*, Dose-dependent stimulation of *KITLG* expression in LX-2 cells by 24-h treatment with the class I-II HDAC inhibitor SAHA (n=3/group). *D*, Dose-dependent inhibition of the SB415286-induced stimulation of *KITLG* expression by the p300/PCAF HAT inhibitor garcinol (24 h) in LX-2 cells (n=3/group). Drugs were applied following 24-h serum deprivation. Groups marked by asterisk are different from the control group, and groups not sharing the same superscript letter are different from each other (*P*<0.05 by post-hoc multiple comparisons).

We performed ChIP-PCR targeting the core *Kitl* promoter in murine gastric smooth muscles exposed for 6 h to rhIGF1 or SB415286 using antibodies against several activating and repressive histone marks and the polycomb group (PcG) HKMT EZH2. Control experiments using “non-immune” mouse IgG and antibodies against the functionally irrelevant Gapdh protein revealed little recovery of *Kitl* promoter DNA and no variation among treatments (Figure S5). The biological role of the enzymes and other proteins involved in the establishment of the targeted modifications was then validated with the aid of pharmacological inhibitors and RNAi in LX-2 and GIST-T1 cells using *KITLG* expression as readout. We first studied the role of histone acetylation, which is almost invariably associated with transcriptional activation [46] (Figure 5). In murine gastric smooth muscles, both rhIGF-I and SB415286 increased the occupancy of the *Kitl* promoter by H4ac (which may include acetylated K5, K8, K12 and K18 [47]) (Figure 5A), whereas increased occupancy by H3K9ac was only detected in response to GSK3i (Figure 5B). Consistent with these findings, inhibition of HDAC classes I-II with SAHA led to a dose-dependent increase in *KITLG* mRNA in LX-2 cells (Figure 5C). The role of increased H3K9 acetylation in the GSK3i effect was supported by the dose-dependent reduction of SB415286-induced increase in *KITLG* mRNA by the p300/PCAF HAT inhibitor garcinol (Figure 5D). In GIST-T1 cells, the transcriptional effects of HDAC inhibition could not be determined due to rapid cell death likely reflecting the apoptotic effects of acetylation and consequent loss of function of the KIT chaperone heat shock protein 90 [48].

**Figure 6:**
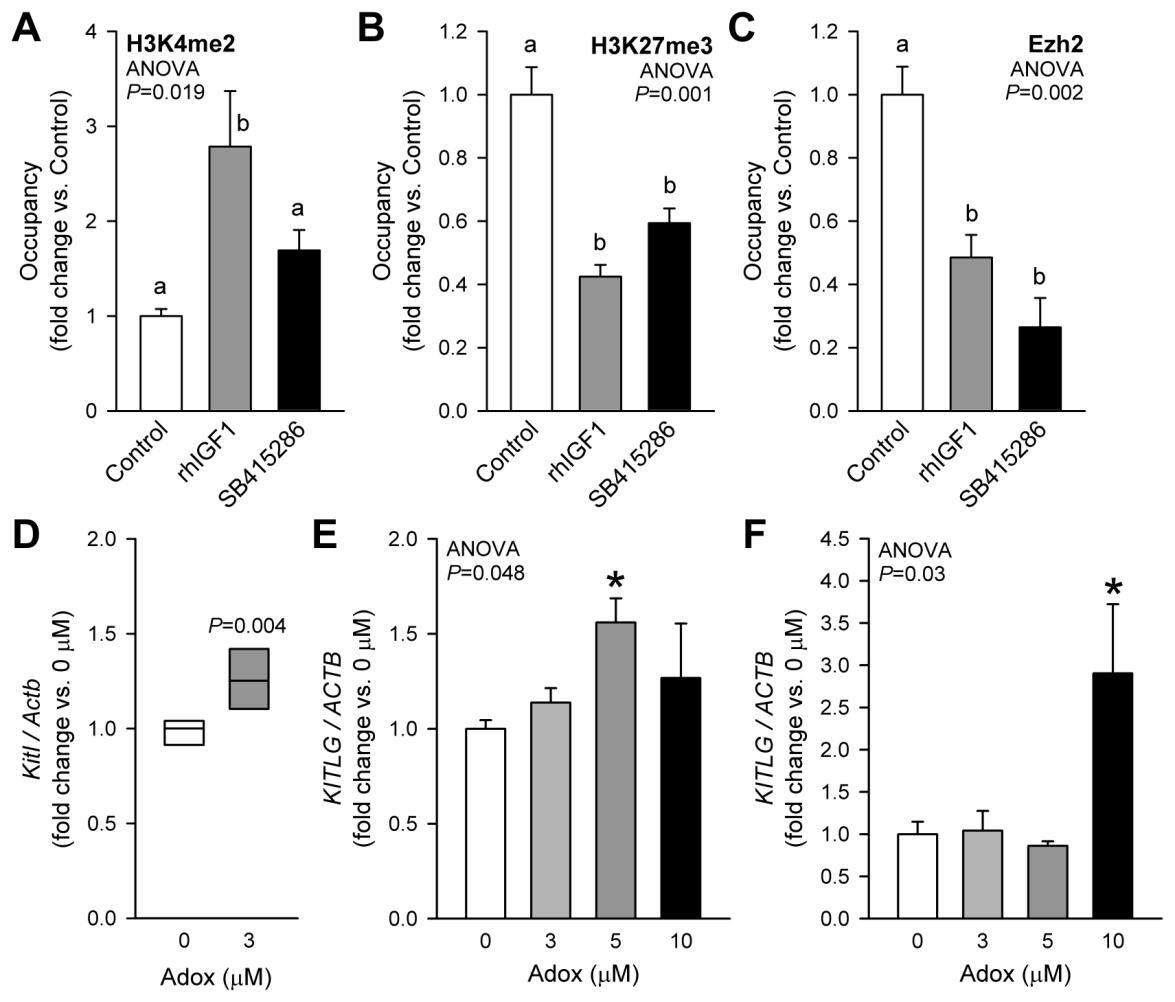
Role of trithorax- and polycomb-mediated histone modifications in IGF1- and GSK3i-induced activation of *Kitl/KITLG* transcription. *A*, Increased occupancy of the *Kitl* core promoter by the trithorax group-mediated, activating H3K4me2 histone modification in response to 6-h rhIGF1 (100 ng/mL) and SB415286 (30 µM) treatment in murine gastric smooth muscles. *B*-*C*, Reduced occupancy of the *Kitl* core promoter by the PRC2-mediated, repressive H3K27me3 histone modification (*B*) and by the PRC2 histone methyltransferase EZH2 (*C*) in response to rhIGF1 and SB415286 in the same tissues. *D*-*F*, Stimulation of *KITLG* expression by the indirect histone methyltransferase inhibitor Adox in murine gastric smooth muscles (*D*), LX-2 cells (*E*) and GIST-T1 cells (*F*) (n=3/group). Adox was applied for 72 h following 24-h serum deprivation. See further details in the legend to Figure 5.

Next, we examined whether histone methylation, a biochemical mechanism associated with long-term transcriptional memory, also contributes to the regulation of *Kitl/KITLG* expression. First we studied the activating H3K4me2 mark, which binds enhancer and promoter regions and gene bodies of actively transcribed or transcriptionally poised but repressed, tissue-specific genes [49]. Whereas rhIGF1 increased the level of H3K4me2 on the *Kitl* promoter ~2.8-fold, SB415286 only had a modest effect (Figure 6A). Since transcriptional activation mediated by H3K4 methylation is often antagonized by PcG complexes [50,51], we also measured the association with the *Kitl* promoter of the PcG HKMT EZH2 and the repressive H3K27me3 modification it catalyzes [50]. These experiments showed that rhIGF1 and SB415286 reduced the presence of both EZH2 and the H3K27me3 mark on the *Kitl* promoter (Figure 6B-C). To mechanistically explore the functional impact of the PcG-mediated repression on *Kitl/KITLG* expression, we inhibited EZH2 activity with the indirect HKMT inhibitor Adox [29]. Adox increased *KITLG* mRNA in GIST-T1 cells ~2.9-fold (Figure 6F) but had more modest, albeit statistically significant, effects in murine gastric smooth muscles and LX-2 cells (Figure 6D-E). These results indicate that PcG inhibits *Kitl/KITLG* expression under basal conditions.

**Figure 7:**
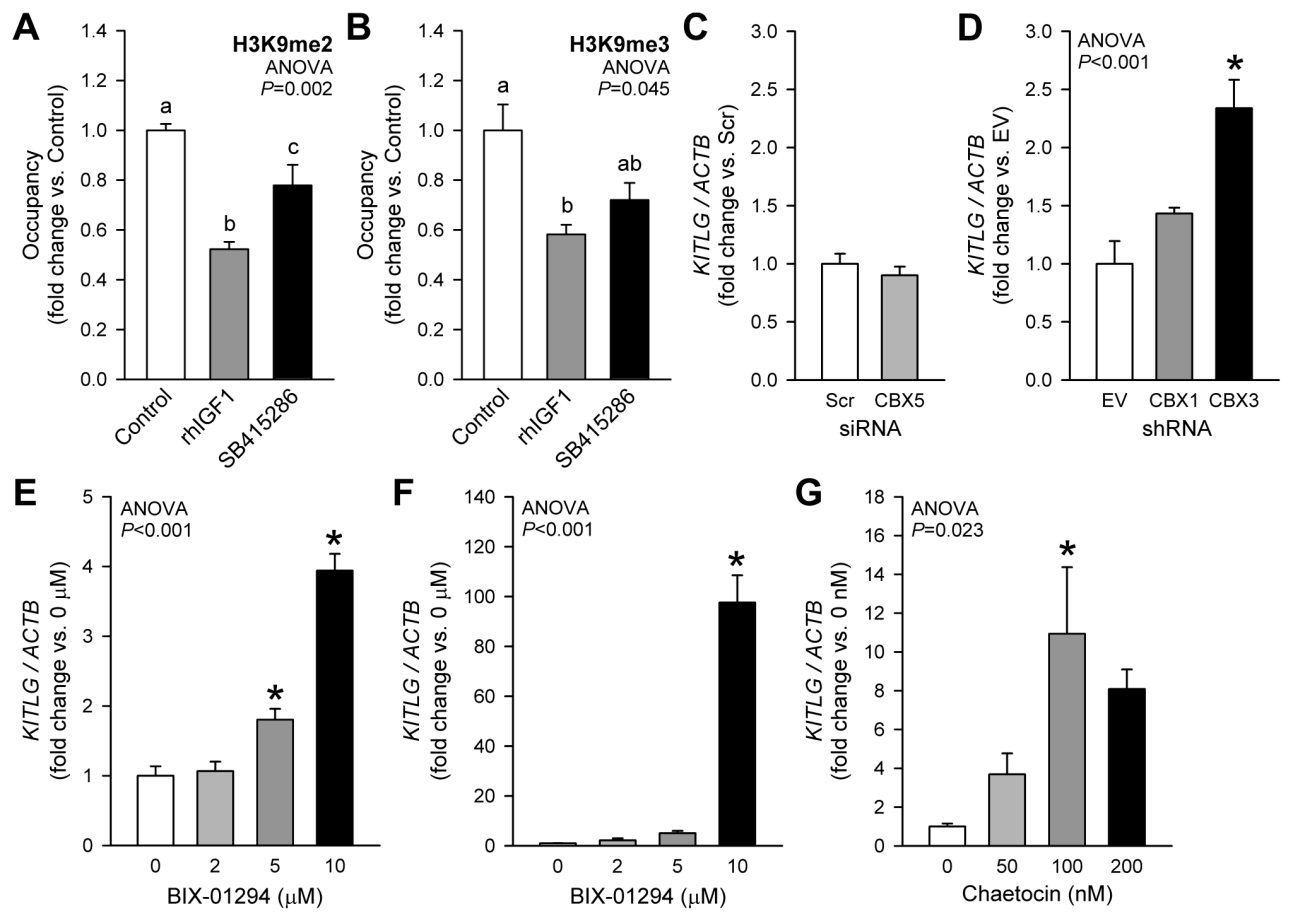
Role of reduced H3K9 methylation in the activation of *Kitl/KITLG* transcription by IGF1 and GSK3i. *A*-*B*, Reduced occupancy of the *Kitl* core promoter by the repressive H3K9me2 (*A*) and H3K9me3 (*B*) histone modifications in response to 6-h rhIGF1 (100 ng/mL) and SB415286 (30 µM) treatment in murine gastric smooth muscles. *C*-*D*, Probing the role of HP1 isoforms in transcriptional repression of *KITLG* in LX-2 cells by siRNA- (CBX5: HP1α; 25 nM, 72 h; *C*) or shRNA-mediated knock-down (CBX1: HP1β; CBX3: HP1γ; 30 µg plasmid, 72 h; *D*). Note activation of *KITLG* expression by shRNA-mediated knock-down of HP1γ (n=3/group). *E*-*F*, Stimulation of *KITLG* expression by the G9A/GLP H3K9me1/2 HKMT inhibitor BIX-01294 in LX-2 cells (*E*) and GIST-T1 cells (*F*) (n=3/group). *G*, Stimulation of *KITLG* expression by the H3K9 HKMT inhibitor chaetocin in LX-2 cells (n=3/group). Drugs were applied for 24 h following 24-h serum deprivation. See further details in the legend to Figure 5.

We also studied the involvement H3K9 methylation in the regulation of *Kitl/KITLG* transcription. The best understood function of H3K9me2/3 is stable gene silencing through heterochromatin formation [52]. This process requires the binding of HP1 proteins and consequent recruitment of the H3K9me1/2 HKMTs G9A and GLP and the H3K9me3 HKMT SUV39H1, which reinforce the silencing of genes located within the constitutive heterochomatin and initiate the repression of genes previously embedded in euchromatin [53]. In murine gastric muscles, we detected both H3K9me2 and H3K9me3 on the *Kitl* promoter (Figure 7A-B), and both marks were reduced by rhIGF1. GSK3i had less pronounced effects. In LX-2 cells, *KITLG* mRNA increased in response to RNAi targeting HP1γ (CBX3) but was unaffected by HP1α (CBX5) or HP1β (CBX1) knockdown (Figure 7C-D). These findings are consistent with HP1γ’s role in euchromatic silencing [53]. In response to the G9A/GLP inhibitor BIX-01294 we also detected a dose-dependent increase in *KITLG* mRNA in LX-2 cells, and a ~100-fold increase in GIST-T1 cells (Figure 7E-F). In LX-2 cells, *KITLG* expression was also dose-dependently stimulated by chaetocin, a specific inhibitor of SUV39H1 (Figure 7G). The transcriptional effects of chaetocin could not be assessed in GIST-T1 cells due severe cytotoxicity. These results reveal an important role for HP1γ and H3K9me2/3 in IGF1-mediated *Kitl/KITLG* expression.

In summary, our data support the model that IGF1 promotes autocrine/paracrine Kitl/KITLG**–**KIT signaling in part via AKT-mediated GSK3i and coordinated chromatin modifications favoring increased *Kitl/KITLG* transcription, and that these effects can be uncoupled from the direct mitogenic actions of IGF1. To allow *Kitl/KITLG* expression, IGF target cells must increase the levels of activating H4ac, H3K9ac and H3K4me2 marks and decrease the levels of the repressive marks H3K27me3 and H3K9me2/3. While IGF1 and GSK3i appeared to exert their effects via the same general mechanisms, they also showed differences in their actions. The results presented herein outline a novel RTK cross-talk that regulates ligand-induced KIT activation in both physiologic/developmental and oncogenic contexts.

## Discussion

Adequate stimulation of the KIT receptor by its ligand Kitl/KITLG is required for diverse developmental and physiological processes such as hematopoiesis, pigmentation, gametogenesis, spatial learning, cardiomyocyte differentiation and repair, vasculogenesis and angiogenesis, lung function and gastrointestinal motility [1,2]. In contrast, ligand-independent activation of KIT due to oncogenic mutations is associated with several types of cancer such as GIST, seminomas, acute myeloid leukemia, melanomas and systemic mastocytosis [1,6]. However, abnormal ligand-dependent signaling occurring in the absence of mutations and reflecting altered expression of KIT and/or Kitl/KITLG contributes to the pathogenesis of not only non-neoplastic disorders such as gastrointestinal dysmotilities and allergies [1,21,22] but also various cancers including subsets of GIST, acute myeloid leukemia, small-cell lung carcinoma, breast and colorectal cancer, ovarian cancer and neuroblastoma [1,9,11-13]. Therefore, understanding the factors and mechanisms regulating Kitl/KITLG expression will likely provide novel therapeutic tools for both functional disorders and cancers. We have previously shown that long-term IGF1 treatment of intact murine gastric *tunica muscularis* increases Kitl availability in part by stimulating the growth and survival of smooth muscle cells, the primary source of Kitl in this tissue [22]. Here we demonstrate that IGF1 directly stimulates Kitl/KITLG expression in gastrointestinal smooth muscles, the natural microenvironment for ICC and GIST [8,21,22], in GIST cells and in LX-2 cells derived from hepatic stellate cells [33], the presumed niche for KIT^+^ hepatic progenitors [54], inflammatory cells [55] and hepatocellular carcinoma [25].

Our study outlines a transcriptionally mediated autocrine/paracrine loop between two distinct RTK signaling systems, the Kitl/KITLG**–**KIT and IGF**–**IGF1R systems. Locally produced and circulating IGF1 affects almost all tissues and plays key roles in the regulation of body size, skeletal acquisition, muscle mass, reproduction, metabolism and life/health span [56]. There is also a strong positive correlation between circulating IGF levels and cancer risk and prognosis [14,15]. IGF1R signaling facilitates cell cycle progression mainly at the G_1_-S transition reflecting increased cyclin D1 transcription and translation stimulated via the ERK MAPK and AKT**–**MTOR**–**p70S6K pathways, respectively [15]. As our data show, increased IGF1R activation (in part from autocrine IGF1; Figure S1G) can, in turn, increase Kitl/KITLG expression and thus potentially amplify its oncogenic potential in KIT-expressing cells and tissues. For example, in WT GIST, an IGF2**-**IGF1R autocrine/paracrine loop sustained by overexpression of both the receptor and its ligand in the same tumor microenvironment [5,16,17,57,58] may activate a secondary autocrine/paracrine loop formed by KITLG and WT KIT leading to increased KIT phosphorylation (refs. 9,10,13 and present results) and cell proliferation. Indeed, our data in the heterozygous *KIT* mutant GIST-T1 cells indicate that autocrine/paracrine KITLG-mediated KIT signaling may account for ~40% of baseline proliferation even in cells with a secondary, imatinib-resistant mutation and can be inhibited with antibodies or RNAi targeting KITLG. Since imatinib preferentially targets mutant receptors [6,9], inhibiting the IGF–IGF1R and KITLG–KIT coupled autocrine/paracrine loops may be beneficial in GIST, which are in most cases heterozygous for a given *KIT* mutation and continue expressing WT KIT protein [5]. However, the contribution of this pathway to GIST growth in vivo remains to be established. Interestingly, even in GIST lacking WT KIT, anti-KIT antibodies inhibited tumor growth with an efficacy similar to what we observed in GIST-T1 cells following KITLG immunoneutralization, although the former effect was attributed to increased phagocytosis and stimulation of KIT degradation rather than prevention of KITLG binding [59]. In hepatic stellate cells, KITLG expression stimulated by IGF1R signaling may facilitate the KIT-dependent recruitment of inflammatory cells in injury and fibrosis [55] and progenitor cells in hepatic failure [54], and may thus be associated with both disease progression and tissue regeneration. In gastrointestinal neuromuscular tissues, stimulation of Kitl/KITLG**–**KIT signaling could prevent or counter ICC loss associated with several motility disorders and conditions including diabetic gastroparesis [22,60] and aging [43,61], where reduced Kitl/KITLG expression is a pathogenetic factor [22,43]. Since ICC differentiation cannot be supported solely by supplying free Kitl/KITLG [2,21], pharmacological interventions will likely require stimulation of local production of both soluble and membrane-associated Kitl/KITLG, which, as our data show, could be accomplished by IGF1 administration or GSK3i. In summary, the crosstalk between the IGF and the Kitl/KITLG pathways revealed by our results is potentially important for understanding and controlling the mechanisms of cell proliferation in both neoplastic and functional gastrointestinal diseases such as GIST, liver disease and gastroparesis. Therefore, it is also important to discuss the information we have obtained on the pathway mediating signal transduction from the IGF1R to the nucleus to regulate Kitl/KITLG expression.

Our initial studies focused on the intracellular kinases that transduce signals downstream of IGF1R. In our models, Kitl/KITLG expression could also be stimulated by inhibiting GSK-3 activity with both ATP-competitive and non-ATP-competitive inhibitors. These results were unexpected because IGF1 effects on Kitl appeared to involve overlapping actions of several major pathways including the ERK MAPK, AKT and MTOR**–**p70S6K pathways, whereas GSK3i stimulated Kitl expression without increasing ERK1/2 or p70S6K phosphorylation and cyclin D1 expression at time points when IGF1 effects on these parameters were prominent. The potential translational significance of these findings is that GSK3i may allow the stimulation of KITLG expression e.g. in patients with ICC loss without concomitant activation of the pathways that mediate IGF1’s actions promoting cellular stress, aging and cancer [14,15]. While GSK3i may affect ERK1/2 and MTOR**–**p70S6K at other time points, our results agree with Kuemmerle’s finding that in gastrointestinal smooth muscle cells, the AKT**–**GSK3i-mediated and p70S6K- and ERK1/2-dependent mechanisms are functionally segregated, with AKT-dependent signaling being involved only in anti-apoptotic but not in the proliferation-stimulating actions of IGF1 [19,20]. Indeed, despite mediating the β-catenin-stabilizing effect of WNT signaling, long-term GSK3i has been associated with reduced, rather than increased, cancer risk possibly due to the concomitant activation of forkhead transcription factors [28], and numerous GSK-3 inhibitors are under investigation for their beneficial effects in diabetes, inflammation, central nervous system injuries, bipolar disorder, Alzheimer’s disease and cancer [45].

Subsequently, we investigated how the IGF1- and GSK3i-induced signaling activates nuclear events to bring about increased Kitl/KITLG expression. By measuring *Kitl/KITLG* transcript levels and *KITLG* promoter activity we found that Kitl/KITLG protein expression induced by IGF1 or GSK3i indeed reflected increased gene transcription. However, our data also revealed differences in the time-courses of the IGF1 and GSK3i effects along with preferential stimulation by IGF1 and GSK3i in GIST-T1 and LX-2 cells, respectively. Therefore, we studied the role of several repressive and activating histone modifications, as well as the enzymes and regulatory factors responsible for their establishment by ChIP and pharmacologic and RNAi-mediated interventions. In selecting our ChIP targets we considered that both the mouse and human *Kitl/KITLG* promoter contain a CpG island but are unlikely to be DNA-methylated, given that we detected significant RNA polymerase II occupancy, baseline expression and transcriptional activity in our models. We found that activation of *Kitl* expression in murine gastric smooth muscles by both IGF1 and GSK3i elicited coordinated changes in the chromatin involving reduced and increased occupancy of the *Kitl* promoter by repressive and activating histone marks, respectively. These events involved the activating histone marks H4ac, H3K9ac, H3K4me2, the repressive marks H3K9me2/3 and H3K27me3, as well as related enzymes and regulators such as EZH2, G9A, GLP, SUV39H1, PCAF, HDACs and HP1γ. Our data also indicated some differences between IGF1 and GSK3i-induced changes such as preference for H3K4me2- and H3K9ac-mediated activation by IGF1 and GSK3i, respectively. The complexity of this system raises the question how these changes are orchestrated. There is strong evidence that the process of transcription itself regulates chromatin states [52], and sequence-specific transcription factors, besides transiently modulating transcription, contribute to the recruitment of chromatin modifiers [62]. For example, transcription factors activated in response to IGF1 signaling or GSK3i may bind to the promoter and initiate the recruitment and eviction of the chromatin modifying complexes that establish the activating and repressive marks, respectively [63]. IGF1 may activate transcription via ERK and p70S6K signaling [15], by promoting IGF1R β subunit nuclear translocation and association with lymphoid enhancer-binding factor 1 [23] and via GSK3i. Physiological or pharmacological GSK3i can in turn lead to the loss of inhibitory phosphorylation of several transcription factors in a stimulus-, promoter- and cellular context-dependent manner [45]. Indeed, some of these GSK-3-inhibited transcription factors have putative or verified binding sites in the promoter region of the *KITLG* gene [1]. Identification of the key transcription factors mediating the observed IGF1 and GSK3i effects on *Kitl/KITLG* expression is a requisite next step toward understanding the transcriptional mechanisms that couple IGF1-IGF1R and Kitl/KITLG–KIT RTK signaling in physiologic and oncogenic contexts.

## Supporting Information

Figure S1 **Kitl/KITLG and IGF1R protein expression and IGF1 secretion** **in murine gastric smooth muscles, human LX-2 stellate cells and GIST cells**. *A*, Detection of Kitl in the lysate of gastric corpus+antrum muscles from a juvenile BALB/c mouse. The membrane was simultaneously probed with antibodies against Kitl and Gapdh (loading control) and appropriate fluorescent secondary antibodies. Note primary Kitl band at ~31 kDa and a weaker band at ~43 kDa. The 21-kDa Kitl band was only borderline detectable. *B*, In human GIST cell lines, only the 31-kDa KITLG band was detected. *B*, Validation of the Western immunoblotting method. The 31-kDa Kitl band was also detected in lysates of *Sl/Sl* ^4^ hematopoietic stromal cells expressing mKitl^248^. No Kitl bands were detected in *Sl/Sl* ^4^ stromal cells lacking full-length Kitl. *D*, Expression of KITLG protein in LX-2 human hepatic stellate cells and three human GIST cell lines. *E*, Expression of KIT protein in the same cell lines. The level reported for LX-2 cells represents background fluorescence as no specific band was detected. Note lack of correlation between KITLG and KIT expression. *F*, IGF1R α and β chain expression in GIST48B cells. *G*, IGF1 secretion into culture media by GIST-T1, GIST882, GIST48B and LX-2 cells. 3000 cells/well were plated into 96-well plates and cultured in the presence or absence of FBS (GIST-T1, GIST882, LX-2: 10%; GIST48B: 15%) for 48 h. IGF1 was measured in the harvested cell-free media using the Quantikine Human IGF1 Immunoassay kit (DG100, R&D systems). Note differential responses to FBS.
(TIF)

Figure S2 **Kitl protein expression is stimulated by IGF1.** Stimulation of Kitl expression, detected using Actb as reference, by 18-h treatment with 100 ng/mL rhIGF1 in gastric corpus+antrum *tunica* *muscularis* organotypic cultures from 14-16-day-old BALB/c mice (n=3/group). Kitl and Actb were simultaneously detected in the same samples by two-color immunofluorescence. Representative immunoblots show identical areas of the blots imaged at different wavelengths. The degree of Kitl upregulation was statistically indistinguishable from the increase detected using Gapdh as loading control (1.89±0.21-fold vs. 2.07±0.15-fold, *P*=0.583).
(TIF)

Figure S3 **SB216763 stimulates Kitl expression without activating cyclin D1 expression and p70S6K and ERK1/2 phosphorylation.** GSK3α/β inhibitor SB216763 was applied to organotypic cultures of gastric corpus+antrum muscles from of 14-16-day-old BALB/c mice at 3 µM. *A*, Effect of 18-h application of SB216763 on Kitl expression; n=5/group. *B*, Effect of the same treatment on cyclin D1 expression; n=3/group. *C*, Effects of 1-h exposure to SB216763 on p70S6K and ERK1/2 phosphorylation; n=3/group. See Figure 2 for further details.
(TIF)

Figure S4 **TDZD-8 stimulates Kitl expression without activating cyclin D1 expression and p70S6K and ERK1/2 phosphorylation.** The non-ATP-competitive GSK3α/β inhibitor TDZD-8 was applied to organotypic cultures of gastric corpus+antrum muscles from of 14-16-day-old BALB/c mice at 10 µM. *A*, Effect of 18-h application of TDZD-8 on Kitl expression; n=6/group. *B*, Effect of the same treatment on cyclin D1 expression; n=3/group. *C*, Effects of 1-h exposure to SB216763 on p70S6K and ERK1/2 phosphorylation; n=3/group. See Figure 2 for further details.
(TIF)

Figure S5 **Negative controls for the ChIP experiments.** *A*, Low recovery of *Kitl* promoter DNA by ChIP performed in murine gastric smooth muscles with non-immune mouse IgG (mIgG) or anti-Gapdh antibody relative to input chromatin or ChIP with anti-RNA polymerase II (RNA pol II) antibody. *B*-*C*, Unchanged recovery of *Kitl* promoter sequence in response to 6-h rhIGF1 (100 ng/mL) and SB415286 (30 µM) treatment in murine gastric smooth muscles following ChIP using mIgG (*B*) or anti-Gapdh antibody (*C*). Representatives of two independent ChIP experiments, each performed in triplicates, are shown.
(TIF)

Table S1 **Antibodies used in Western immunoblotting studies.**
(PDF)

Table S2 **Antibodies used in chromatin immunoprecipitation (ChIP) studies.**
(PDF)
