# Supplementary figures and images for: Membrane-To-Nucleus Signaling Links Insulin-Like Growth Factor-1- and Stem Cell Factor-Activated Pathways
Source: PLoS One. 2013 Oct 7;8(10):e76822. doi: 10.1371/journal.pone.0076822 (PMC3792098; doi:10.1371/journal.pone.0076822)

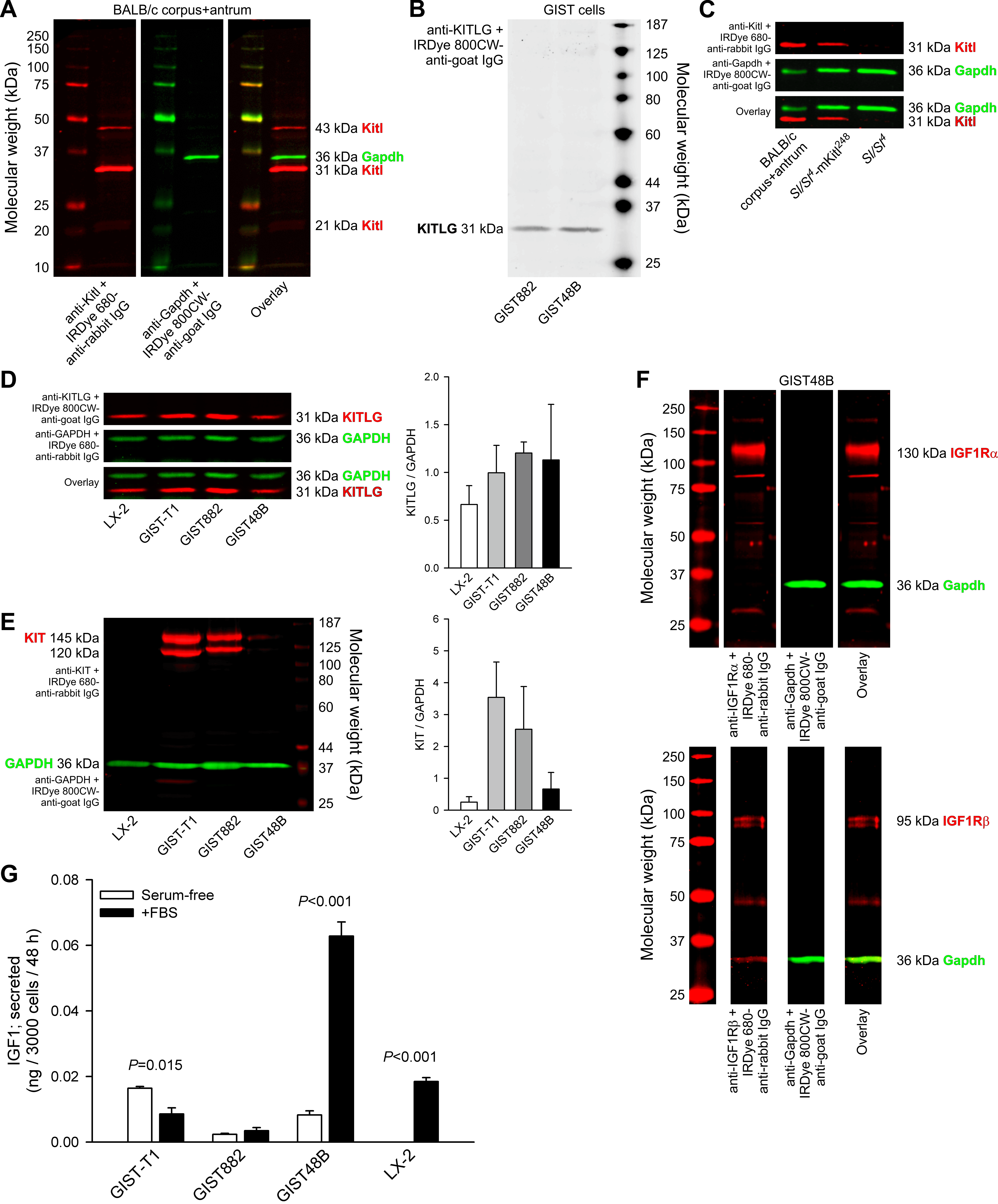

Supplement: Figure S1 — Kitl/KITLG and IGF1R protein expression and IGF1 secretion in murine gastric smooth muscles, human LX-2 stellate cells and GIST cells. A, Detection of Kitl in the lysate of gastric corpus+antrum muscles from a juvenile BALB/c mouse. The membrane was simultaneously probed with antibodies against Kitl and Gapdh (loading control) and appropriate fluorescent secondary antibodies. Note primary Kitl band at ~31 kDa and a weaker band at ~43 kDa. The 21-kDa Kitl band was only borderline detectable. B, In human GIST cell lines, only the 31-kDa KITLG band was detected. B, Validation of the Western immunoblotting method. The 31-kDa Kitl band was also detected in lysates of Sl/Sl 4 hematopoietic stromal cells expressing mKitl248. No Kitl bands were detected in Sl/Sl 4 stromal cells lacking full-length Kitl. D, Expression of KITLG protein in LX-2 human hepatic stellate cells and three human GIST cell lines. E, Expression of KIT protein in the same cell lines. The level reported for LX-2 cells represents background fluorescence as no specific band was detected. Note lack of correlation between KITLG and KIT expression. F, IGF1R α and β chain expression in GIST48B cells. G, IGF1 secretion into culture media by GIST-T1, GIST882, GIST48B and LX-2 cells. 3000 cells/well were plated into 96-well plates and cultured in the presence or absence of FBS (GIST-T1, GIST882, LX-2: 10%; GIST48B: 15%) for 48 h. IGF1 was measured in the harvested cell-free media using the Quantikine Human IGF1 Immunoassay kit (DG100, R&D systems). Note differential responses to FBS. (TIF) [file pone.0076822.s001.tif]

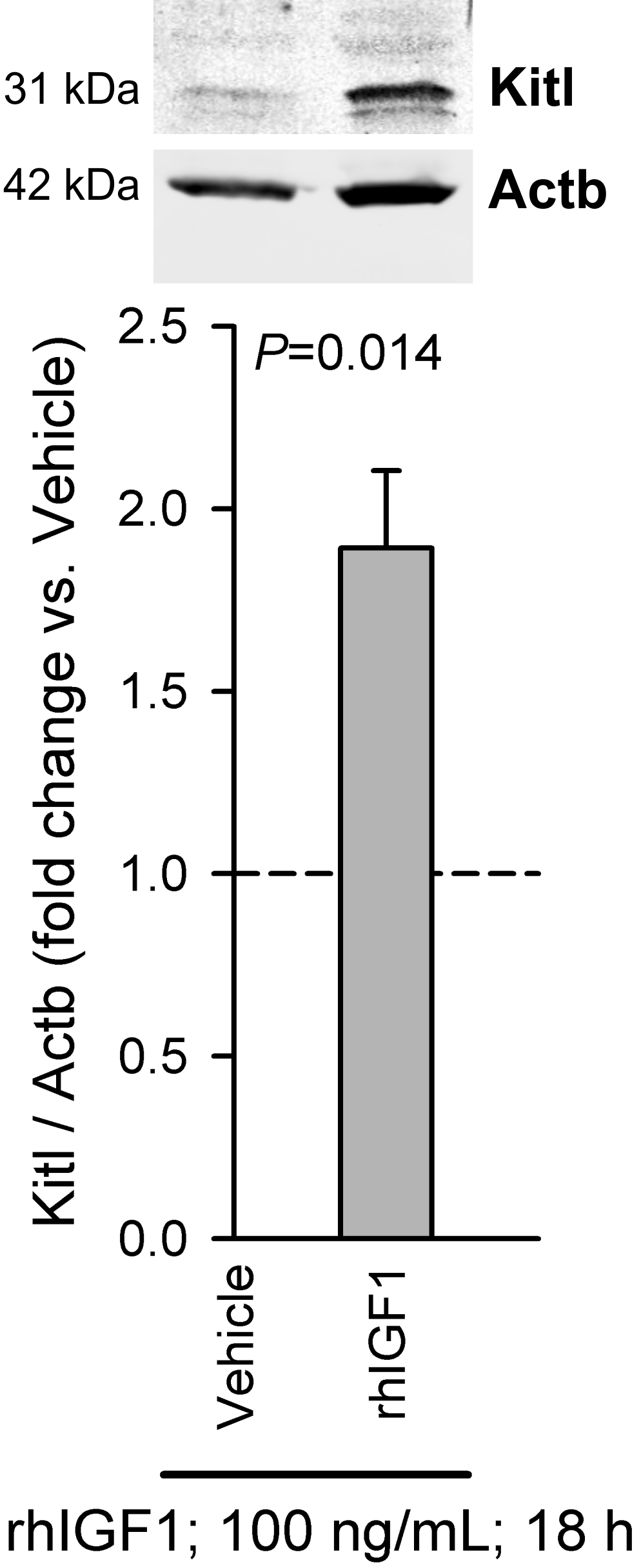

Supplement: Figure S2 — Kitl protein expression is stimulated by IGF1. Stimulation of Kitl expression, detected using Actb as reference, by 18-h treatment with 100 ng/mL rhIGF1 in gastric corpus+antrum tunica muscularis organotypic cultures from 14-16-day-old BALB/c mice (n=3/group). Kitl and Actb were simultaneously detected in the same samples by two-color immunofluorescence. Representative immunoblots show identical areas of the blots imaged at different wavelengths. The degree of Kitl upregulation was statistically indistinguishable from the increase detected using Gapdh as loading control (1.89±0.21-fold vs. 2.07±0.15-fold, P=0.583). (TIF) [file pone.0076822.s002.tif]

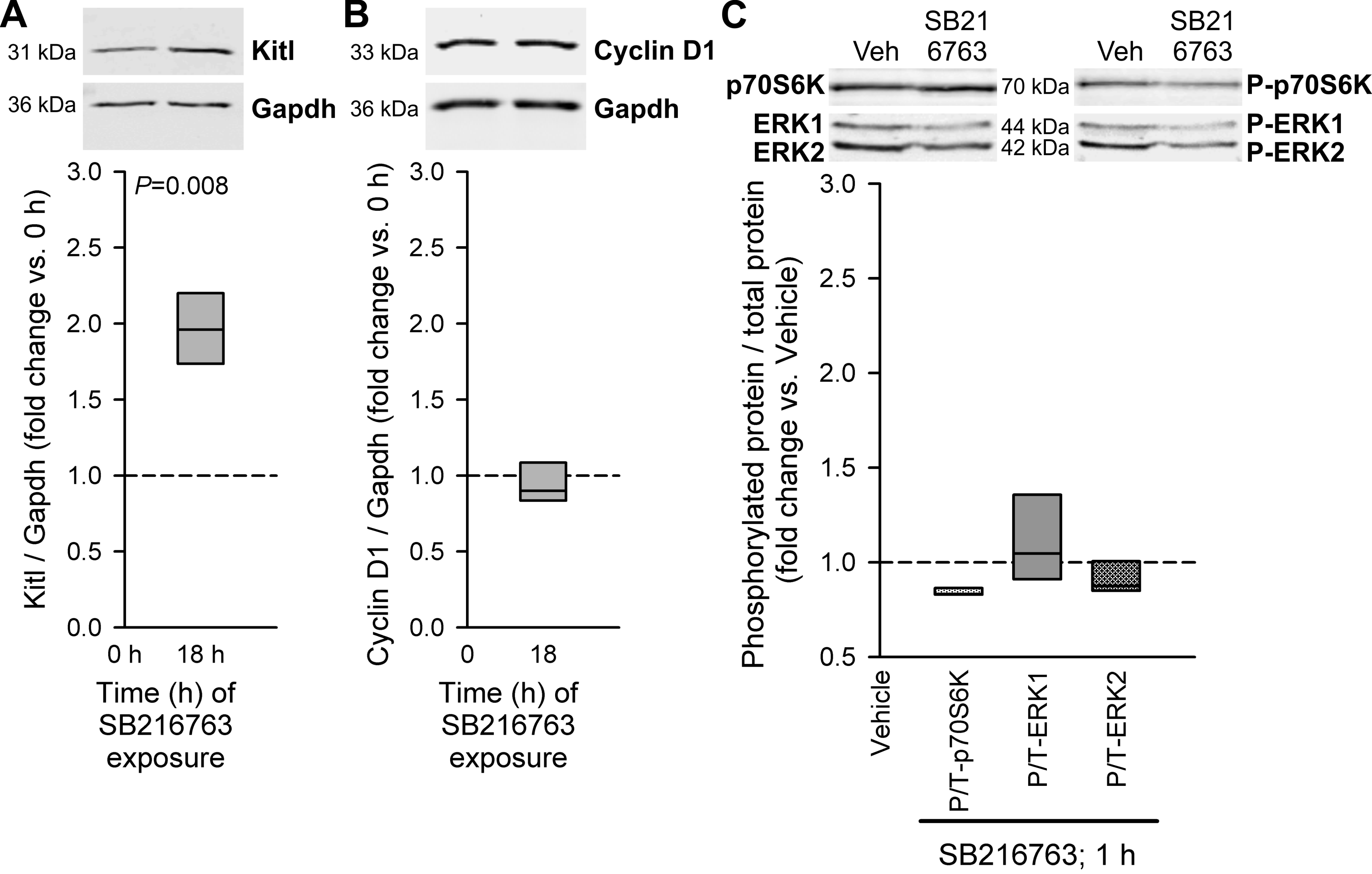

Supplement: Figure S3 — SB216763 stimulates Kitl expression without activating cyclin D1 expression and p70S6K and ERK1/2 phosphorylation. GSK3α/β inhibitor SB216763 was applied to organotypic cultures of gastric corpus+antrum muscles from of 14-16-day-old BALB/c mice at 3 µM. A, Effect of 18-h application of SB216763 on Kitl expression; n=5/group. B, Effect of the same treatment on cyclin D1 expression; n=3/group. C, Effects of 1-h exposure to SB216763 on p70S6K and ERK1/2 phosphorylation; n=3/group. See Figure 2 for further details. (TIF) [file pone.0076822.s003.tif]

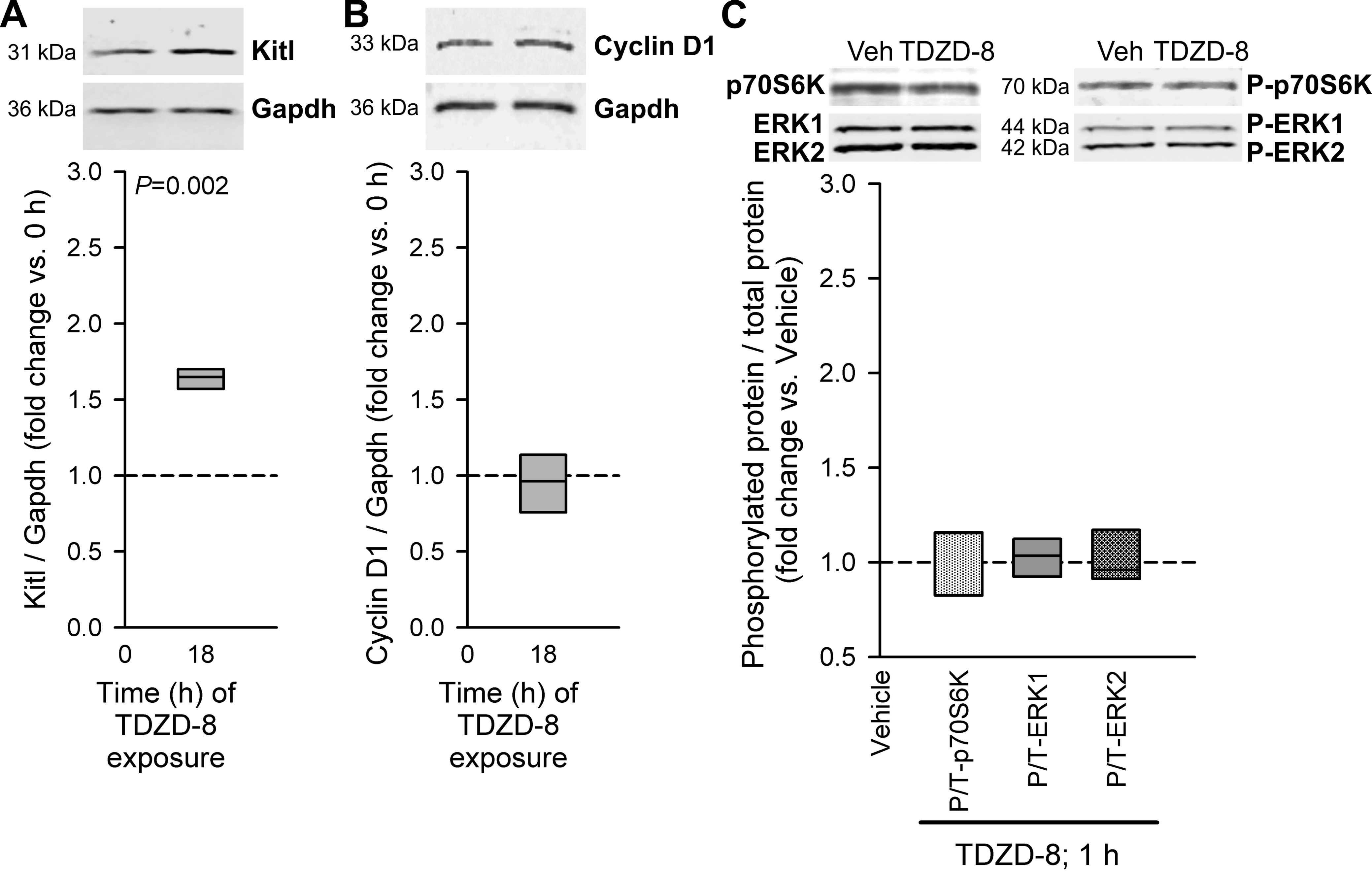

Supplement: Figure S4 — TDZD-8 stimulates Kitl expression without activating cyclin D1 expression and p70S6K and ERK1/2 phosphorylation. The non-ATP-competitive GSK3α/β inhibitor TDZD-8 was applied to organotypic cultures of gastric corpus+antrum muscles from of 14-16-day-old BALB/c mice at 10 µM. A, Effect of 18-h application of TDZD-8 on Kitl expression; n=6/group. B, Effect of the same treatment on cyclin D1 expression; n=3/group. C, Effects of 1-h exposure to SB216763 on p70S6K and ERK1/2 phosphorylation; n=3/group. See Figure 2 for further details. (TIF) [file pone.0076822.s004.tif]

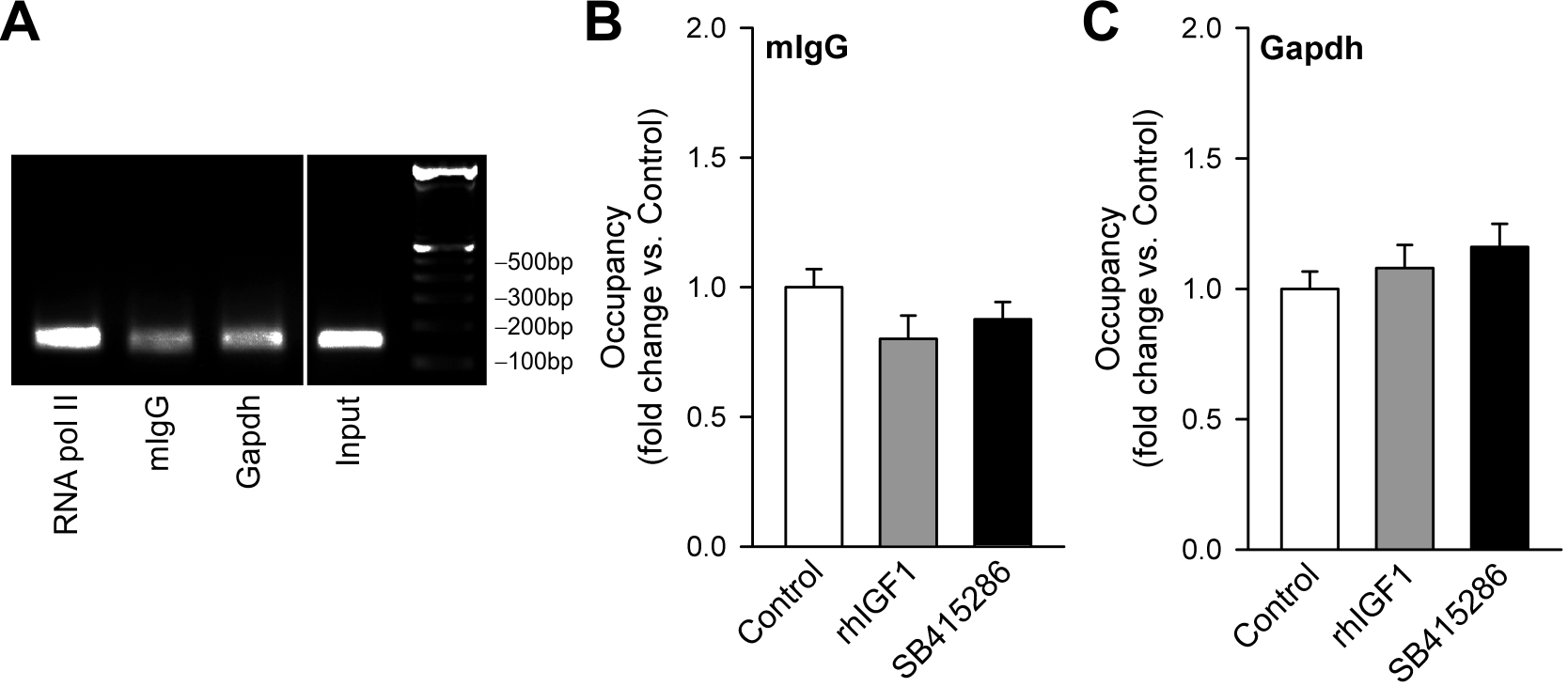

Supplement: Figure S5 — Negative controls for the ChIP experiments. A, Low recovery of Kitl promoter DNA by ChIP performed in murine gastric smooth muscles with non-immune mouse IgG (mIgG) or anti-Gapdh antibody relative to input chromatin or ChIP with anti-RNA polymerase II (RNA pol II) antibody. B-C, Unchanged recovery of Kitl promoter sequence in response to 6-h rhIGF1 (100 ng/mL) and SB415286 (30 µM) treatment in murine gastric smooth muscles following ChIP using mIgG (B) or anti-Gapdh antibody (C). Representatives of two independent ChIP experiments, each performed in triplicates, are shown. (TIF) [file pone.0076822.s005.tif]
