# Supplementary material for: Membrane-To-Nucleus Signaling Links Insulin-Like Growth Factor-1- and Stem Cell Factor-Activated Pathways
Source: PLoS One. 2013 Oct 7;8(10):e76822. doi: 10.1371/journal.pone.0076822 (PMC3792098; doi:10.1371/journal.pone.0076822)
Supplement: Table S1 — Antibodies used in Western immunoblotting studies. (PDF) [file pone.0076822.s006.pdf]

Table S1. Antibodies used in Western immunoblotting studies

Target	Supplier	Host	Clone/ID	Isotype	Label	Final conc.
GAPDH	Imgenex[a]	Goat pAb[b]	IMG-3073			0.05 μg/mL
GAPDH	Sigma-Aldrich[c]	Rabbit pAb	G9545			1:40000
ACTB	BioLegend[d]	Mouse mAb[e]	2F1-1	IgG_{2b}, κ		1:2000
Kitl	AbD Serotec[f]	Rabbit pAb	AAM50	IgG		0.2 μg/mL
KITLG	R&D Systems[g]	Goat pAb	AB-255-NA	IgG		200 ng/mL
KIT	Dako[h]	Rabbit pAb	A4502			1:4000
KIT (C-14)	SCBT[i]	Goat pAb	Sc-1493	IgG		0.2 μg/mL
P-KIT (Y719[j])	CST[k]	Rabbit pAb	#3391			1:2000
IGF1Rα	SCBT	Rabbit pAb	sc-712			1:1000
IGF1Rβ	CST	Rabbit pAb	#3027			1:2000
AKT1/2/3	CST	Rabbit pAb	#9272			1:1000
P-AKT1/2/3 (S473/S474/S472)	BD[l]	Mouse mAb	104A282	IgG_1		1 μg/mL
p70S6K	BD	Mouse mAb	16/p70[s6k]	IgG_1		0.25 μg/mL
P-p70S6K (T389)	CST	Rabbit pAb	#9205			1:1000
ERK1/2	CST	Mouse mAb	3A7	IgG_1		1:4000
P-ERK1/2 (T202/Y204 and T185/Y187)	CST	Rabbit mAb	197G2	IgG		1:1500
GSK3β	BD	Mouse mAb	7/GSK-3b	IgG_1		0.05 μg/mL
P-GSK3β (S9)	CST	Rabbit pAb	#9336	IgG		1:1000
Cyclin D1	CST	Rabbit pAb	#2922	IgG		1:1000
Anti-rabbit IgG (H+L)[m]	LI-COR[n]	Donkey pAb	#926-32223		IRDye 680	1:10000
Anti-mouse IgG (H+L)	LI-COR	Goat pAb	#926-32220		IRDye 680	1:10000
Anti-rabbit IgG (H+L)	LI-COR	Goat pAb	#926-32211		IRDye 800CW	1:10000
Anti-goat IgG (H+L)	LI-COR	Donkey pAb	#926-32214		IRDye 800CW	1:10000

[a]Imgenex Corp., San Diego, CA; [b]pAb, polyclonal antibody; [c]Sigma-Aldrich, Inc., St. Louis, MO; [d]BioLegend, Inc., San Diego, CA; [e]mAb, monoclonal antibody; [f]AbD Serotec, Raleigh, NC; [g]R&D Systems, Inc., Minneapolis, MN; [h]Dako North America, Inc., Carpinteria, CA; [i]SCBT, Santa Cruz Biotechnology, Inc., Dallas, TX; [j]Y719 in mouse KIT corresponds to Y721 in human KIT; [k]CST, Cell Signaling Technology, Inc., Beverly, MA; [l]BD, Becton, Dickinson and Co., Franklin Lakes, NJ; [m]H+L, highly cross-adsorbed ; [n]LI-COR Biosciences, Lincoln, NE.
