# Supplementary material for: Membrane-To-Nucleus Signaling Links Insulin-Like Growth Factor-1- and Stem Cell Factor-Activated Pathways
Source: PLoS One. 2013 Oct 7;8(10):e76822. doi: 10.1371/journal.pone.0076822 (PMC3792098; doi:10.1371/journal.pone.0076822)
Supplement: Table S2 — Antibodies used in chromatin immunoprecipitation (ChIP) studies. (PDF) [file pone.0076822.s007.pdf]

Table S2. Antibodies used in chromatin immunoprecipitation (ChIP) studies

Target	Supplier	Host	Clone/ID	Isotype	Final conc.
EZH2	CST	Mouse mAb	AC22	IgG_1, κ	1:100
H3K27me3	CST	Rabbit mAb	C36B11	IgG	1:100
H3K4me2	Abcam[a]	Rabbit mAb	Y47	IgG	1:500
H3K9me2	Abcam	Mouse mAb	ab1220	IgG_{2a}, κ	1:500
H3K9me3	CST	Rabbit pAb	#9754		1:50
H3K9ac	Millipore[b]	Rabbit pAb	06-942	IgG	1:500
H4ac	Millipore	Rabbit pAb	06-866	IgG	1:500
RNA polymerase II	Millipore	Mouse mAb	CTD4H8	IgG_1	2 μg/mL
GAPDH	Imgenex	Goat PAb	IMG-3073		4 μg/mL

[a]Abcam plc, Cambridge, MA; [b]EMD Millipore Corp., Billerica, MA
